# The Structure of the Large-Scale Heliosphere as Seen by Current Models

**DOI:** 10.1007/s11214-022-00902-6

**Published:** 2022-05-31

**Authors:** Jens Kleimann, Konstantinos Dialynas, Federico Fraternale, André Galli, Jacob Heerikhuisen, Vladislav Izmodenov, Marc Kornbleuth, Merav Opher, Nikolai Pogorelov

**Affiliations:** 1 Theoretische Physik IV, Ruhr-Universität Bochum, 44780 Bochum, Germany; 2 Office of Space Research and Technology, Academy of Athens, 10679 Athens, Greece; 3 Center for Space Plasma and Aeronomic Research, University of Alabama in Huntsville, Huntsville, AL 35899 USA; 4 University of Bern, Bern, Switzerland; 5 Department of Mathematics and Statistics, University of Waikato, Hamilton, 3240 New Zealand; 6 Moscow Center of Fundamental and Applied Mathematics, Lomonosov Moscow State University, Moscow, Russia; 7 Space Research Institute (IKI) of Russian Academy of Sciences, Moscow, Russia; 8 Astronomy Department, Boston University, Boston, MA 02215 USA; 9 Radcliffe Institute for Advanced Study at Harvard University, Cambridge, MA USA

**Keywords:** Heliosphere, Analytical, Numerical, Modeling

## Abstract

This review summarizes the current state of research aiming at a description of the global heliosphere using both analytical and numerical modeling efforts, particularly in view of the overall plasma/neutral flow and magnetic field structure, and its relation to energetic neutral atoms. Being part of a larger volume on current heliospheric research, it also lays out a number of key concepts and describes several classic, though still relevant early works on the topic. Regarding numerical simulations, emphasis is put on magnetohydrodynamic (MHD), multi-fluid, kinetic-MHD, and hybrid modeling frameworks. Finally, open issues relating to the physical relevance of so-called “croissant” models of the heliosphere, as well as the general (dis)agreement of model predictions with observations are highlighted and critically discussed.

## Introduction

The study of the heliosphere, the bubble of hot plasma that the Sun’s wind continuously carves into the surrounding interstellar medium (ISM), started only some six decades ago with the discovery of the solar wind (SW) itself by the Luna-1 and Mariner spacecraft and the ensuing theoretical considerations by E. Parker, V.B. Baranov and others relating to the interaction of this wind with the ISM. As an astronomical topic, the outer heliosphere is unique in that it marks the most distant region of space that can still be observed in situ, most notably by the two spacecrafts *Voyager 1 (V1)* and *Voyager 1 (V1)* (see Richardson et al. 2022; Dialynas et al. 2022), which were only recently complemented by the *New Horizons* probe. The scientific exploration of the outer heliosphere has been, and continues to be, a highly successful joint effort of observational campaigns (both in situ and remote, as with, e.g. the *IBEX* and *IMAP* space observatories at $\sim1~\text{au}$, together with *Cassini* at $\sim10~\text{au}$), theoretical concepts, and increasingly sophisticated numerical simulations. Many theories and concepts had to be revised (and some discarded) along the way, and many open questions remain. This review paper makes an effort to highlight some of the methods and results that have been employed to model the large-scale heliosphere and to settle some of these questions (and has, in doing so, often paved the way to other, novel questions and exciting ideas).

The topic to be covered is obviously a vast one, and this review will necessarily remain incomplete. It also naturally reflects the respective views and fields of expertise of the various authors who contributed to it, which has undoubtedly lead to many interesting and relevant works being left unaddressed. As authors, we jointly regret this, and refer the reader to the accompanying chapters of this topical volume, particularly to the papers by Fraternale et al. (2022) on turbulence and Sokół et al. (2022) on the modeling of energetic neutral atoms and pickup ions. Particularly this last one will likely have some partial overlap, and thus suggest itself prominently for further reading.

This paper is organized as follows. After this introduction, Sect. 2 defines and motivates terms and concepts which are of relevance for this paper, and possibly also for the entire topical volume. Sections 3 and 4 reviews past and present analytical and numerical modeling efforts, respectively, aimed at various aspects of the large-scale heliosphere, followed by chapters on three major simulation frameworks, namely the UAH Multi-Scale Fluid-Kinetic Simulation Suite (MS-FLUKSS), the Moscow model, and the Boston (BU) model in Sects. 5, 6, and 7, respectively. The topical, and at this point still somewhat controversial, issue of split-tail models is addressed in a separate Sect. 8. Finally, Sect. 9 addresses learned lessons and open questions arising from the comparison of modeling results to observations, and Sect. 10 concludes the paper with a summary.

## Important Concepts and Terminology

### Separator Surfaces

At its most basic level, the heliosphere is classically defined as the circumsolar region of space which is influenced by the solar wind (SW), a thermally driven plasma flow emanating radially away from the Sun that was first modeled by Parker (1958) in a now classic paper. Since the Sun travels through the local interstellar medium (LISM) at a speed of approximately $26~\text{km}/\text{s}$ (e.g. Wood et al. 2015), this motion induces a second, largely homogeneous, uni-directional wind as seen in the Sun’s rest frame. The interface between both wind flows is called the heliopause, a surface separating the hot, dilute SW plasma from the colder plasma of the LISM. Starting from the Sun at sub-sonic speeds, the SW becomes supersonic inside 10 solar radii ($R_{\odot}$) and approaches an approximately constant speed of about $400\text{--}700~\text{km}/\text{s}$ at larger distances from the Sun. Beyond about 10 au, the SW is subject to a gradual deceleration due to its interaction with interstellar neutral hydrogen (e.g. Holzer 1972; Whang 1998). Additionally, the SW flow has to decelerate further upon approaching the heliopause, to which it is forced to eventually become tangential. This requirement establishes the presence of another closed shock surface, the termination shock (TS), across which the SW first becomes subsonic again before being redirected tailwards. On the interstellar side, the corresponding surface would be the bow shock (BS), at which the incoming flow is forced to decelerate before being directed around the heliopause. The existence of the BS depends on the value of the fastest speed of signal propagation (the fast magnetosonic speed), and is subject to current scientific debate (e.g. Ben-Jaffel et al. 2013; Scherer and Fichtner 2014, and references therein), the alternative being a “bow wave” (e.g. McComas et al. 2012).

The terminology for these regions is unfortunately not consistent throughout the literature. Some authors (e.g. Borovikov et al. 2011; Burlaga et al. 2018; Chalov et al. 2016; Fahr et al. 2016; Izmodenov et al. 2014; Röken et al. 2015; Zank et al. 2013) refer to the space enclosed between the TS and the HP as the “inner heliosheath” and the region just beyond the HP as the “outer heliosheath,” in analogy to the sheath found inside the bow shock surface surrounding a supersonically moving body. A second group of authors (e.g. Zank 2016; McComas et al. 2017a; Dialynas et al. 2021) prefers to designate these regions as simply the “heliosheath” (HS, without additional qualifiers) and the “very local interstellar medium” (VLISM), respectively. Confusingly, “heliosheath” and “inner heliosheath” are sometimes even used interchangeably. Throughout this paper, this latter variant, i.e. the heliosheath/VLISM pair of terms, will be employed.

Likewise, the method by which the HP itself is identified seems to vary across publications, with numerical works in particular often using the isosurface of quantities like density or temperature for practical reasons. (See Fig. 2 in Michael et al. 2018 for an illustration of the topologically different shapes obtained from different HP methods.) A less ambiguous, and only slightly more involved method – besides following streamlines, which is only useful in stationary situations – is to rely on passive tracer fluids (as done by, e.g. Röken et al. 2015), i.e. to dynamically follow a scalar quantity that has been assigned constant but distinct values at the respective upstream flow boundaries. This situation suggests that it would seem very beneficial if authors adopted the habit of spelling out which definitions they adhere to in a given publication to avoid misunderstandings. Section 3, for instance, considers the HP to be the surface separating fluid elements by origin, such that a given point is inside the HP if and only if there is a streamline connecting it to the Sun.

### Key Properties of the Magnetized Solar Wind

Since the terminal radial SW speed is considerably higher than the fast magnetosonic speed at which fluid perturbations may travel, the super-fastmagnetosonic region upstream of the TS is causally disconnected from any interstellar influences (safe for those due to incident neutral particles, which enter the inner heliosphere unhampered). Therefore, the heliosphere inside the TS is predominantly shaped by solar influences, most notably its magnetic field, as well as by the influx of interstellar hydrogen, whose number density exceeds that of SW ions beyond about 10 au.

The magnetic field at the solar ‘surface’ is structured on a multitude of spatial and temporal scales. Of these, the variations throughout the solar cycle are certainly of pivotal importance for the magnetic structure of the heliosphere. The global photospheric field is observed to alternate between a dipolar configuration, whose axis of symmetry is approximately coincident with the axis of solar rotation, prevailing during solar minimum, and a more irregular distribution of magnetic flux without discernible symmetry during solar maximum. The polarity reverses during the eleven-year period between consecutive minima (Schwabe cycle), only returning to the same polarity after a full Hale cycle of $T_{\mathrm{H}}\approx 22$ years.

Close to the Sun, the global minimum field is largely dominated by an equatorial belt of so-called helmet streamers, and a set of more radial field lines near the polar regions, a setting that was first numerically modeled by Pneuman and Kopp (1971). This region has a plasma beta $\beta < 1$, such that the Lorentz force forces the plasma to corotate with the Sun. But as soon as the magnetic energy density drops below the kinetic energy density of the wind at the so-called Alfvén surface, this varying distribution of radial flux at the Sun’s surface causes field lines to be drawn out more radially by the flow of hot, ionized plasma.

As a result of the interplay between solar rotation and radial advection (see Sect. 3.1), field lines then generally assume the form of Archimedian spirals on cones of constant heliolatitude, while the polarity separator gives rise to an equatorial current sheet. But since even the minimum field is not of exact axial symmetry, this current sheet is typically of a wavy, spiral-like nature, with noticeable excursions in heliolatitude that intensify further towards solar maximum. In addition to the periodic changes in the number of observable sunspots, by which the solar cycle was first established and its minima/maxima phases defined, this cycle manifests itself also in a latitudinal variation of the SW flow speed. During solar minimum, observations (e.g. McComas et al. 2003) indicate a clear dichotomy of ‘slow’ ($350\text{--}500~\text{km}/\text{s}$ at Earth orbit) and ‘fast’ ($\sim 750~\text{km}/\text{s}$) wind emanating from the equatorial streamer and the polar regions, respectively, while the solar maximum phase is characterized by a much more irregular, mostly faster wind. Because of the combination with the above-mentioned departure from axial symmetry, which is strong except for occasional periods during solar minimum, the radial SW flow speed may often vary noticeably along a given heliolatitude circle. This gives rise to alternating waves of fast and slow radial streams that lead to regions of either compression or rarefaction of gas which, in the inertial frame, assume the shape of interwoven density spirals. These corotating interactions regions (CIRs, see, e.g. Kopp et al. 2017, and references therein), which may even form shock fronts, can merge into global merged interaction regions, which may remain detectable even in the outer heliosphere.

A separate class of non-periodic transients are solar flares and coronal mass ejections (CMEs), large eruptions on the photosphere that travel through interplanetary space at high speeds, sometimes interacting with planetary magnetospheres. These will not be covered further in this chapter, though more details on these may be found, e.g., in the recent reviews by Archontis and Syntelis (2019), Temmer (2021), Riley et al. (2018), and references given therein.

## Analytical Models

### Solar Wind in the Inner Heliosphere

Historically the first, and therefore understandably the most basic theoretical model of the solar wind was developed by Parker (1958). It assumes a compressible, isothermal (i.e. with an adiabatic exponent of $\gamma =1$), unmagnetized, purely radial outflow, for which a speed profile $u(r)$ satisfying the ordinary differential equation

1 $$ \frac{u^{2}-c^{2}}{u} \frac{\mathrm{d}u}{\mathrm{d}r} = \frac{2 c^{2}}{r} - \frac{GM_{\odot}}{r^{2}} $$

may be deduced. Here, $G$ is the gravitational constant, $M_{\odot}$ the mass of the Sun, and $c$ is the constant isothermal sound speed of order $\sim130~\text{km}/\text{s}$ for a coronal temperature of 1 MK. When discarding both purely supersonic winds and purely subsonic ‘breeze’ solutions, as well as accretion flows (Bondi 1952) as unphysical, the only reasonable remaining solution to Equation (1), whose mathematical stability was already investigated by Parker (1966) and, more recently, by Keto (2020), may either be found numerically or be expressed in terms of the Lambert W function (Cranmer 2004). This solution is a wind that monotonously accelerates outwards, becomes supersonic ($u=c$) at the critical (sonic) radius $r_{\mathrm{crit}}:= 2GM_{\odot}/c^{2} \sim 0.1~\text{au}$, and then, formally, tends to the monotonously increasing profile $2c \sqrt{\ln (r/r_{\mathrm{crit}})}$ for distances $r \gg r_{\mathrm{crit}}$. However, the assumption of constant temperature, which at small radii may be justified through the effects of adiabatic cooling compensating heating due to, e.g. waves in the extended corona (for an overview thereof see the recent reviews by Banerjee et al. 2021; De Moortel and Browning 2015, and Cranmer and Winebarger 2019), will break down at such large radii. There, it is therefore more reasonable – and also supported by measurements – to assume the expansion to proceed adiabatically rather than isothermally, resulting in a constant SW speed. This, in turn, implies a density profile $n(r) \propto r^{-2}$ in order to satisfy mass conservation, and, from the adiabatic equation of state, the temperature to decrease as $T(r) \propto r^{2(1-\gamma )} = r^{-4/3}$ for $\gamma =5/3$. The isothermal Parker SW model may be generalized to adiabatic exponents $\gamma \ne 1$ (Parker 1963, Chap. 5), see also Keppens and Goedbloed (1999) and Shivamoggi and Rollins (2019), when accepting implicit expressions also for the sonic radius.

Weber and Davis (1967) used the stationary induction equation of ideal magnetohydrodynamics (MHD)

2 $$ \nabla \times ( \mathbf{u} \times \mathbf{B} ) = \mathbf{0} $$

and the requirement of magnetic solenoidality

3 $$ \nabla \cdot \mathbf{B} = 0 $$

to further generalize the Parker wind to a nonzero magnetic flux at a reference sphere (which is typically, though not necessarily, taken to be either the photosphere or the coronal base), but in doing so had to restrict themselves to the equatorial plane. The inclusion of a back-reaction of the Lorentz force close to the Sun establishes a corotation zone within the Alfvén radius, in which field lines act as a lever arm removing angular momentum from the rotating Sun. This further induces the occurrence of two additional critical radii for the other characteristic speeds, as a result of which the solution topology in $(u,r)$ space attains a more complicated form. The still further generalization to all heliolatitudes (e.g. Sakurai 1985) was only possible through a fully numerical treatment.

The shape of the magnetic field lines resulting from a given distribution of flux density $B_{0}(\vartheta ,\varphi _{0})$ as a function of angular position $(\vartheta ,\varphi _{0})$ at radius $r=b$ and with the streamline’s footpoint coordinate $\varphi _{0}$ given by

4 $$ \frac{r}{b} -1 -\ln \left ( \frac{r}{b} \right ) = \frac{u_{\mathrm{sw}}}{b \Omega _{\odot}}(\varphi -\varphi _{0}) $$

was also worked out by Parker in his 1958 paper, where it is derived from Equations (2) and (3) as

5 $$ \mathbf{B}(r,\vartheta ,\varphi ) = B_{0}(\vartheta ,\varphi _{0}) \left (\frac{b}{r}\right )^{2} \left [ \mathbf{e}_{r} - \frac{(r-b) \Omega _{\odot}\sin \vartheta}{u_{\mathrm{sw}}} \, \mathbf{e}_{\varphi}\right ] $$

for a radial flow $\mathbf{u}$ of constant magnitude $u_{\mathrm{sw}}$, and $\Omega _{\odot}$ the angular rotation frequency of the Sun. As opposed to the Weber and Davis (1967) model, the solution expressed in Equation (5) is only valid in the weak-field limit ($\beta \gg 1$), in which field lines are passively advected and do not exert any back-reaction on the flow. For this reason, the source radius $b$ must be chosen to lie beyond the Alfvén radius.

It should be noted that many authors (e.g. Owens and Forsyth 2013; Lhotka and Narita 2019) do not refer to the general form of Equation (5), but rather its frequently used special case of constant $B_{0}$ in the limit $r \gg b$, to wit

6 $$ \mathbf{B}(r,\vartheta ,\varphi ) = B_{0} \left (\frac{b}{r}\right )^{2} \left [ \mathbf{e}_{r} - \frac{r \, \Omega _{\odot}\sin \vartheta}{u_{\mathrm{sw}}} \, \mathbf{e}_{\varphi}\right ] $$

as the ‘Parker spiral field’, with the latter authors even (wrongly) criticizing Parker’s model for not recognizing the sign reversal of the dipolar magnetic field across the two hemispheres. This sign reversal, however, is easily included by prescribing, say, $B_{0}(\vartheta ,\varphi _{0}) \propto \cos \vartheta /|\cos \vartheta | \in \{\pm 1\}$, and Parker (1958) actually does mention $B_{0}(\vartheta , \varphi _{0}) \propto \cos \vartheta $ as a possible choice to represent the solar dipole. In fact, several “generalizations” of the Parker spiral field, like that to a nonzero tilt angle (Kota and Jokipii 1983), can be understood as simply choosing a particular form of $B_{0}(\vartheta ,\varphi _{0})$. Several other, more phenomenological approaches, like the one by Lhotka et al. (2016) with its custom radial dependence of the normal component, suffer from not satisfying the solenoidality constraint (3).

For applications in the outer heliosphere, the near-Sun variations mentioned above can be safely neglected. What cannot be ignored, however, are time-dependent effects because typical fluid crossing times are generally (much) larger than $T_{\mathrm{H}}$, the duration of the Hale cycle. (What exactly constitutes a “typical crossing time” obviously depends on the extent and position of the region under investigation. Taking 50 km/s as the flow speed in the heliotail (e.g. Müller et al. 2008), the crossing time for a distance of, say, 1000 au is almost 100 yrs, and even longer in the opposite direction towards the upwind stagnation point.) The incorporation of solar-cycle effects by a simple $\cos (2\pi \, t /T_{\mathrm{H}})$ factor, as done by, e.g. Kocifaj et al. (2006), is clearly admissible only locally because of its instantaneous effect, ramping the global field up and down in sync. The correct way to introduce a realistic global time dependence would be through a time-dependent boundary condition at the solar source surface and a subsequent radial propagation using

7 $$ \partial _{t} \mathbf{B} = \nabla \times ( \mathbf{u} \times\mathbf{B} ) , $$

the time-dependent version of the induction equation (2) It is vital to observe that in this situation, the solenoidality condition (3) poses non-trivial constraints on the set of admissible boundary conditions (see also Röken et al. 2021). For instance, simply multiplying the boundary field $B_{0}(\vartheta ,\varphi _{0})$ by a factor like $\cos (\omega t)$ to emulate a solar cycle of period $2\pi /\omega $ will in general cause the entire region $r>b$ to be swamped by a divergence-laden, and hence unphysical, magnetic field topology.

### Beyond the Termination Shock

A frequently used model for the flow in and around the heliosphere is that of the Rankine half-body^1^

8 $$ \frac{\mathbf{u}}{u_{0}} = -\nabla \left (\frac{q}{r} + z \right ) = \left ( \frac{q}{r^{2}} - \cos \vartheta \right ) \mathbf{e}_{r} + ( \sin \vartheta ) \mathbf{e}_{\vartheta } $$

first proposed and discussed in the heliospheric context by Parker (1961) and used thereafter by many authors (e.g. Yu 1974; Fahr et al. 2014; Röken et al. 2015; Sylla and Fichtner 2015; Galli et al. 2019). It consists of the superposition of a uniform flow $u_{0}$ incident from the $+z$ direction and a point source of strength $4\pi u_{0} q$ at the origin. Both flows are separated by a heliopause-like surface given by

9 $$ z_{\mathrm{hp}}(\rho ) = \frac{2-\rho ^{2}}{\sqrt{4-\rho ^{2}}} \quad \text{and} \quad r_{\mathrm{hp}}(\vartheta ) = \frac{1}{\cos (\vartheta /2)} $$

in cylindrical and spherical coordinates, normalized to the upwind stand-off distance $\sqrt{q}$. At large tailward distances, this surface tends to a semi-infinite cylinder of radius $\rho =2\sqrt{q}$.

Since the Rankine flow (8) derives from the gradient of a flow potential, it cannot include discontinuities like shock surfaces, and the undisturbed radial SW and homogeneous ISM flows are only attained asymptotically. However, Senanayake and Florinski (2013) were able to generalize the flow to include a spherical, rather than point-like source surface, inside of which a purely radial SW may be prescribed. This property of the boundary sphere, which has to be centered on the Sun to warrant mass conservation, is suggestive of its use to represent the TS, although simulations (e.g. Izmodenov 2000; Müller et al. 2008) typically show a prolate TS, with the downwind distance to the Sun being about twice as large as the upwind distance. Earlier, Nerney and Suess (1995) had presented a further (albeit only approximate) extension to potential flow emanating from a mildly non-spherical TS surface to accommodate a latitudinal dependence of SW speed (Phillips et al. 1995) at the TS.

A kinematic MHD solution for the magnetic field in the HS and heliotail can in principle be found by solving either the stationary (2) or time-dependent (7) induction equation for the flow field of Equation (8). Yu (1974) considered this problem for a static, bimodal inner boundary field whose axis of symmetry makes an angle $\alpha $ with the plane perpendicular to the inflow direction (such that the angle between the magnetic symmetry axis and the inflow direction itself is $\pi /2-\alpha $), and derived an approximate expression for the field components, valid in a plane perpendicular to the axis and located at infinite downwind distance. For the solar case ($\alpha =0$), these planar images confirm the notion of two identical lobes of field lines, a Northern and a Southern one, spiraling around a central field lines within a single heliotail, while smaller angles cause one lobe to dominate and the other to transform into an annulus encircling the other lobe’s cross section as $\alpha \rightarrow \pi /2$. The full solution for the general, time-dependent case was recently derived by Röken et al. (2021), though yet without specializing to explicit solar boundary conditions.

The Rankine flow model (8) is particularly useful for situations in which the magnetic field is weak enough to allow for its back-reaction on the flow dynamics to be neglected. For the opposite situation, i.e. a solar/stellar wind expanding into an almost static but strongly magnetized ISM, Parker (1961) derived a HP geometry of an infinite cylinder and a sphere with two polar outflow channels as the respective limits of low and high pressure at infinity, and a smooth transition in between these two extremes.

### Shape Models for the VLISM Magnetic Field

Analytical models for the large-scale magnetic field beyond the heliopause are equally sparse, any may be grouped into two classes. First, the so-called “shape models” work by prescribing a heliopause geometry, and then construct a magnetic field which is both tangential to this surface and tends to the undisturbed homogeneous ISM field far away from this surface, thereby exploiting a concept also used, for instance, to model planetary magnetospheres (e.g. Kobel and Flückiger 1994). (A potential shortcoming of this general approach is the lack of an associated velocity field, which may or may not be a problem for a given application.) Most notably, Schwadron et al. (2014) start with a half-sphere acting as the cap of a semi-infinite cylinder, assume that no currents flow outside this model heliopause ($\nabla \times \mathbf{B}=\mathbf{0}$), and consequently derive their field as the gradient of a scalar potential $\Phi $, which then in turn satisfies a Poisson equation

10 $$ \nabla ^{2} \Phi = \nabla \cdot (\nabla \Phi ) = \nabla \cdot (- \mathbf{B}) = 0 $$

that is solved subject to boundary conditions enforcing parallel field lines at the heliopause. The corresponding field $\mathbf{B}=-\nabla \Phi $ is derived separately for the upwind ($z>0$) and downwind ($z<0$) half-space, and a small current has to be accepted when matching both solutions at the $z=0$ interface.

In an interesting earlier approach, Whang (2010) first also noted that, since the nose of the heliopause is not too dissimilar to a half-sphere, the magnetic field draping around it may qualitatively be approximated by flow lines (or, equivalently, flow-aligned field lines) of the inviscid flow around a sphere of radius $a$. These, in turn, can be written as the superposition of a point dipole and an aligned homogeneous flow as

11 $$ \mathbf{B}_{\mathrm{nose}} = B_{0} \, \mathbf{e}_{z} + \frac{B_{0} a^{2}}{2} \, \nabla f , $$

where

12 $$ f(x,y,z) = \frac{z}{(x^{2}+y^{2}+z^{2})^{3/2}} $$

is the scalar potential of a $z$-aligned dipole of unit strength centered at the origin. The key idea to extend this concept also to the downwind heliopause (i.e. the half-space $x>0$ in the coordinate system used by Whang 2010) is to replace the single dipole by an semi-infinite linear progression of such dipoles of infinitesimal strength, giving

13 $$ \mathbf{B} = \mathbf{B}_{0} + B_{0} \, a^{2} \eta \int _{c}^{\infty}\nabla f(x-x^{\prime},y,z) \, \mathrm{d}x^{\prime} $$

where additionally the ISM field is allowed to have an arbitrary orientation different from $\mathbf{e}_{z}$. Constants $\eta $ and $c$ are adjusted such that the distant tail tends to a straight cylinder of radius $a$. Equation (13) is easily accessible to direct integration, yielding simple and compact expressions for the field components. Specifically, for the case of a flow-parallel field, one obtains $\eta =c/a=1/2$, and the Rankine-type heliopause shape (9) is recovered exactly.

### Kinematic Models for the VLISM Magnetic Field

As a second class of models are those that actually solve the (stationary) ideal induction equation (2) for the magnetic field for a given flow field, which is usually again the Rankine half-body flow of Equation (8). Since the heliopause shape given by Equation (9) is already implicitly contained in this flow field, the only relevant boundary condition is that of the magnetic field at upstream infinity, which is again taken to be homogeneous (but arbitrarily oriented). More importantly, these kinematic MHD models have the additional benefit of being associated with a physical plasma flow field, which, by construction, is fully consistent with the derived magnetic field.

This long-standing problem was only recently addressed independently by Röken et al. (2015) and Isenberg et al. (2015), with both works coincidentally being published contemporaneously in the same journal. The latter work centers on Euler potentials (e.g. Stern 1966) and a decomposition of the advected field into transversal and longitudinal (flow-parallel) contributions, the latter of which is trivially found from being proportional to velocity. Röken et al. (2015), on the other hand, presented two separate derivations of the same result. The first one directly solved the induction equation as a coupled system of partial differential equations, while the second relied on Cauchy’s integral method. It thus observes that streamlines and isochrones (lines of constant travel times) form a non-orthogonal grid of coordinate lines with respect to which the components of the advected magnetic field are constant throughout their entire motion, and hence equal to their values at the boundary.

In both cases, a crucial step is to obtain an expression to quantify the total travel time $T(r,\vartheta )$ of a given fluid element at position $(r,\vartheta )$, which, in spherical coordinates, may be found from either one of the two expressions

14 $$ \int _{\infty}^{r} \frac{\mathrm{d}r^{\prime}}{u_{r}(r^{\prime})|_{\Psi}} = T(r,\vartheta ) = \int _{0}^{\vartheta } \frac{r^{\prime}\, \mathrm{d}\vartheta ^{\prime}}{u_{\vartheta }(\vartheta ^{\prime})|_{\Psi}} $$

that are based on the respective definitions of velocity components $u_{r}$ and $u_{\vartheta }$. It is important to note that in both cases integration has to proceed along a fixed streamline, conveniently identified by ensuring constancy of $\Psi (r^{\prime},\vartheta ^{\prime})$, with

15 $$ \Psi (r,\vartheta ) = 1-\cos \vartheta - \frac{r^{2} \sin ^{2} \vartheta }{2} $$

the Stokes stream function of the Rankine flow. While actual values for the travel times are necessarily infinite, only the differential values of neighboring fluid elements are physically relevant for the computation of the magnetic field components. This can rigorously be dealt with by starting the integration at a finite upwind distance $z_{\mathrm{up}}$, and only later consider the resulting magnetic field components in the limit $z_{\mathrm{up}} \rightarrow \infty $.

**Fig. 1:**
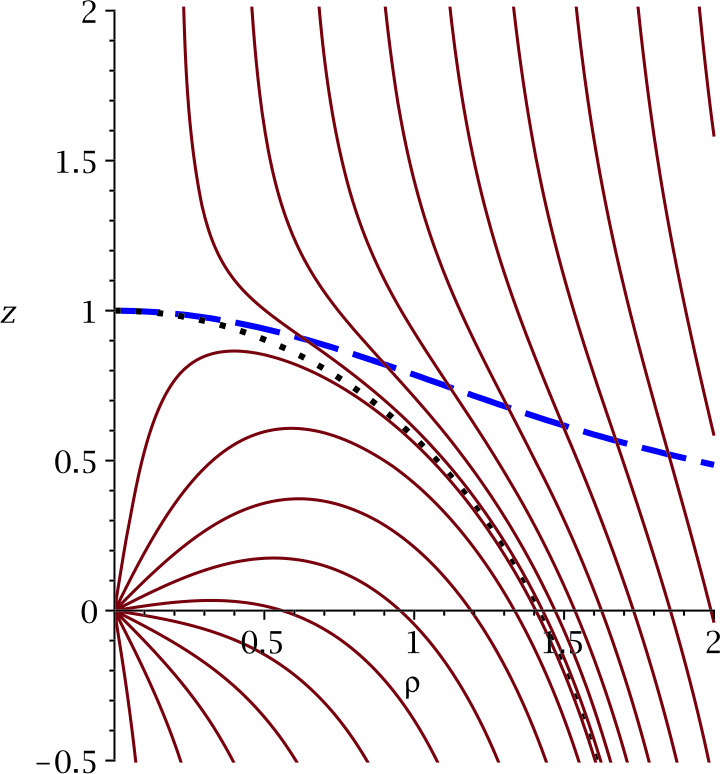
Streamlines of the Rankine half-body flow (solid), both inside and outside the HP (dotted) as given by Equation (9). The blue dashed line indicates the surface at which $u_{r}=0$, corresponding to a fluid element’s locus of closest approach to the Sun as it travels along a given streamline

While both expressions in Equation (14) are equivalent from a mathematical point of view, integrating $1/u_{r}$ radially entails the complication of $u_{r}$ passing through zero (see Fig. 1). For this reason, Isenberg et al. (2015), who used exactly this option, had to bridge the associated coordinate singularity by way of approximation. $u_{\vartheta }$, on the other hand, is non-zero along any streamline, which allowed Röken et al. (2015) to derive an exact analytical solution valid in the entire space exterior to the heliopause without having to rely on any kind of approximations.

Being an exact solution to the ideal, stationary induction equation, the full solution is very similar to its upstream boundary value in both direction and magnitude, and only starts to drape around the HP in the immediate vicinity of the latter. On the HP itself, the field is parallel to the former and reaches infinite field strength. This is an unavoidable consequence of idealness and stationarity, which forces incoming magnetic flux to pile up ahead of the HP indefinitely. In a more realistic, non-ideal setting, an equilibrium between advection and diffusion would cause the field magnitude to remain finite. This could in principle be modeled by adding a resistive term $\propto \nabla \times (\eta \nabla \times \mathbf{B})$ with nonzero resistivity $\eta $ to the RHS of Equation (2). However, the additional complexity of the second-order differential operators and the associated loss of exact field-flow coupling would offer little hope of analytical tractability.

Since the infinitely high magnetic “wall” around the HP is not only nonphysical but also poses practical problems, e.g. for the simulation of cosmic-ray particles who cannot cross this boundary, a viable method to arrive at a finitely-amplified field without compromising either Equation (2) nor (3) was employed by Florinski et al. (2021). The key idea is first to observe that only the transversal field component (which is initially perpendicular to the flow and always parallel to isochrones) diverges, while the longitudinal part tends to zero on the HP. Second, by assigning a freely adjustable factor to each isochrone and then scaling the transversal field by the factor of its isochrone, it is possible to attain an arbitrary field strength profile along the inflow axis, including one that matches observed values. While this modification leaves the validity of both Equation (2) and (3) unchanged, a possibly relevant shortcoming is that the magnetic field no longer tends to the undisturbed boundary field at large crosswind ($\rho \rightarrow \infty$) or downwind ($z \rightarrow -\infty$) distances.

Since the underlying flow field (8) has $\nabla \cdot \mathbf{u}=0$ and therefore does not allow for density variations across flow lines, Kleimann et al. (2017) presented a generalization of the Röken et al. (2015) solution to compressible flow. Introducing the upstream Mach number $m$ as a new parameter (with $m=0$ reproducing the previous incompressible version), a more realistic configuration could be found that retains many properties of the $m=0$ case, such as streamline geometry and the shape of the HP, but now features a finite mass pile-up ahead of the stagnation point, a more gradual increase in upwind field strength, and a generally improved similarity to fully self-consistent MHD simulations of the same setup. It is worth noting that the compressible model retains the base model’s analytical tractability for arbitrary orientation of the boundary field, as well as – by construction – the feature of infinite field strength at the HP. A new kinematic MHD-based model of the magnetic field on both sides of the HP combining the practical benefits of an analytical global field model with a globally finite-valued field magnitude is currently under development (Kleimann 2022, in preparation).

### Distortion Flows

As already stated in Sect. 1, models employing an analytical approach generally have to accept a considerable amount of simplifying assumptions. Even so, the number of known exact MHD solutions is relatively small, and those that are of relevance in the heliospheric context are even smaller. In particular, the cylindrical symmetry of the Rankine flow (8) enforces a circular cross section of the heliopause/-tail. This is in stark disagreement with the notion of the magnetic pressure of the ISM field working to compress the heliotail and elongating its cross section in the perpendicular direction (parallel to the direction of the undisturbed ISM field). This phenomenon also routinely becomes evident in simulations, see for instance the left panel of Fig. 5, or Fig. 6 in Heerikhuisen et al. (2014), Fig. 2 in Izmodenov and Alexashov (2015). In order to accommodate this effect into MHD models of the heliotail, Kleimann et al. (2016) developed and proposed the use of so-called “distortion flows,” by which an existing solution to Equation (2), which may be given analytically or numerically in terms of fields $\mathbf{u}$ and $\mathbf{B}$, can be forced into a different geometrical shape while still satisfying Equations (2) and (3) exactly. A distortion flow $\mathbf{w}$ is a stationary flow field in which both $\mathbf{u}$ *and* $\mathbf{B}$ are passively advected, and it can be shown that any such field satisfying the condition $\nabla (\nabla \cdot \mathbf{w})=\mathbf{0}$ will leave the validity of Equations (2) and (3) unchanged for any pair of fields $[\mathbf{u}, \mathbf{B}]$ advected therein. Specifically, data sets from a heliosphere simulation by Heerikhuisen et al. (2014) were used to derive a heliotail aspect ratio varying as

16 $$ \eta _{\mathrm{fit}}(z) = 1 + 1.37 \left (\frac{-z}{100~\text{au}} \right ) \left (\frac{B_{\mathrm{ism}}}{\text{nT}} \right )^{2.5} $$

with tailward distance $-z$ and ISM field strength, indicating the increasing flattening with both magnetic ISM field strength $B_{\mathrm{ism}}$ and distance $-z$. The specific distortion flow $\mathbf{w}=\alpha (z) x\, \mathbf{e}_{x}-\alpha (z) y\, \mathbf{e}_{y}$, for instance, establishes a mapping

17 $$ [x_{0},y_{0},z_{0}] \mapsto [x_{0} \exp (\alpha (z) t), y_{0} \exp (- \alpha (z) t), z_{0}] $$

as a function of formal “time” $t$, and can thus be used to deform the cylindrical Rankine-type heliopause into a tube whose elliptical cross section attains a variable aspect ratio according to Equation (16) simply by choosing $\alpha (z) t=\eta_{\mathrm{fit}}(z)/2$. This allows the advantages of a fully analytical formula to be combined with a realistic tail geometry.

It should be noted that, since this relatively simple choice of distortion flow does not vanish at large crosswind distances ($\rho \rightarrow \infty $ and $z<0$) but rather grows linearly in magnitude, the resulting distorted fields are very different from the respective pristine ISM fields at these distances. This property, however, will not be a practical limitation for most applications which focus on the closer vicinity, or even the interior, of the heliopause.

## Numerical Modeling

**Fig. 2:**
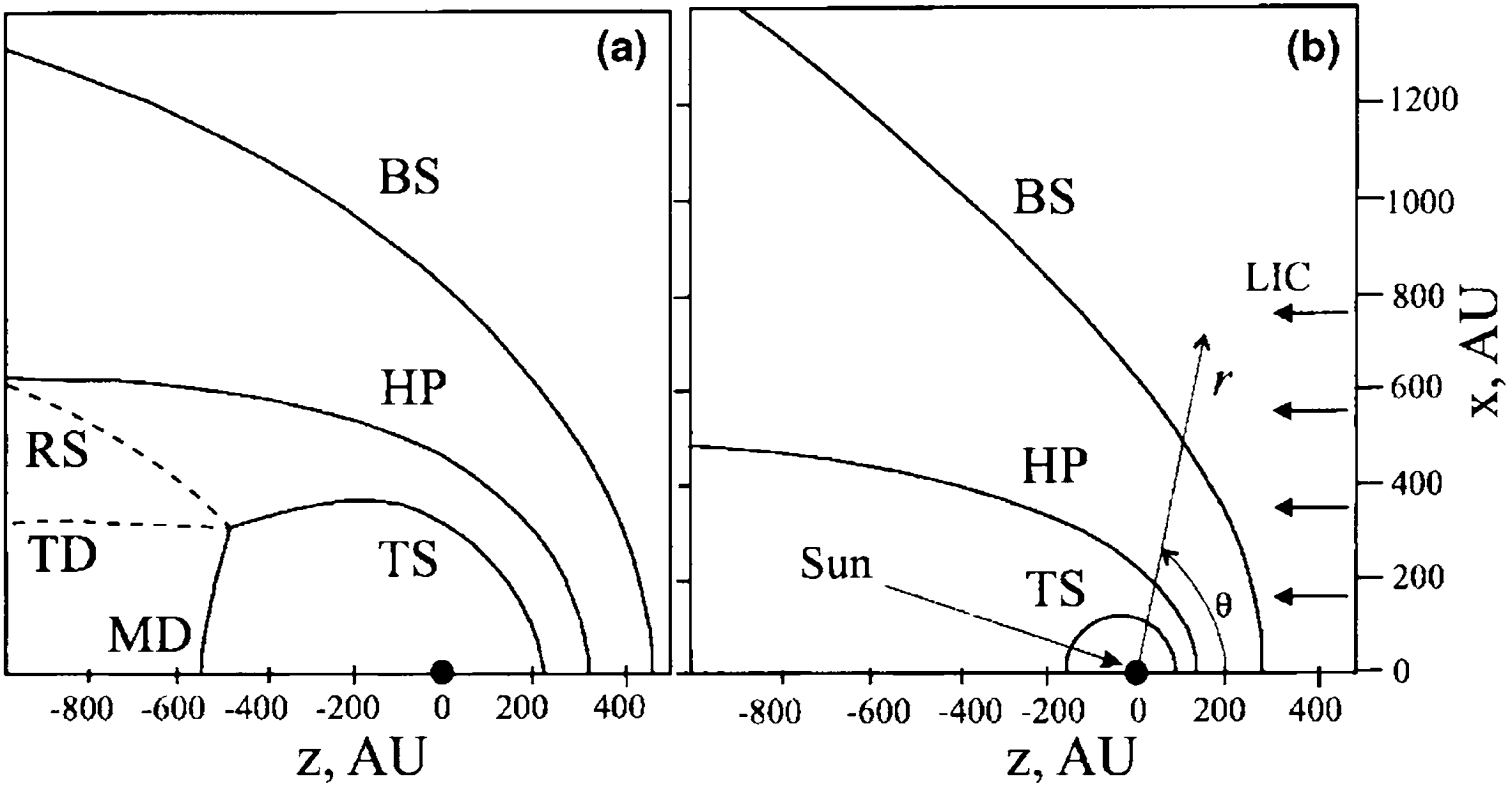
Structure of the SW–LISM interaction region. Left: no H atoms, right: with H atoms taken into account. Adapted from Aleksashov et al. (2004)

Single-fluid simulations of the global heliosphere date back to the work of Baranov et al. (1971) and others, which already reproduced many key features of the hydrodynamic shock structure resulting from the interaction between the supersonic flows of ISM and SW. This includes not only the surfaces mentioned in Sect. 2.1 but also the Mach disk formed by the downwind part of the TS and its boundary, and the triple point, from which both the tangential discontinuity (TD) and a reflected shock (RS) emanate. The latter may get reflected multiple times between the HP and the TD; see left panel of Fig. 2 for an illustration. Since both the SW and LISM magnetic fields exhibit symmetry axes different from that of the LISM flow, the physical realism of full MHD models remained limited until the change from 2D (e.g. Washimi 1993) to fully three-dimensional (3D) grids became computationally feasible. These fully 3D MHD models (e.g. Linde et al. 1998; Pogorelov and Matsuda 1998; Tanaka and Washimi 1999; Izmodenov et al. 2005) were then able to reproduce the symmetry-breaking effect of the LISM field, most notably in the form of a flattening of the heliotail (e.g. Heerikhuisen et al. 2014). As expected, the inclusion of spacecraft data on the SW side tends to yield more complex structures, possibly including a bipolar heliotail (Wood et al. 2014; Izmodenov and Alexashov 2015).

The use of single-fluid models assumes a fully ionized plasma, and has therefore been criticized because the LISM plasma mostly (to a fraction $\sim2/3$) consists of neutral H atoms. Two different approaches have been used to incorporate these into (M)HD models: While the use of multi-fluid models (e.g. Borovikov et al. 2008) remains popular, Baranov and Malama (1993) argued for the need of a kinetic treatment since the mean free path length of neutrals is comparable to the size of the heliosphere. On the other hand, comparisons of both approaches (Alexashov and Izmodenov 2005; Heerikhuisen et al. 2006) identified circumstances in which the resulting differences are rather small, particularly when using multiple neutral populations.

The benchmark-like comparison of different hydrodynamic simulations of the heliosphere by Müller et al. (2008) illustrates and quantifies the degree of disagreement between simulated configurations arrived at using different models/codes that take neutrals into account. Generally speaking, the inclusion of cold, neutral hydrogen from the LISM affects the obtained structures in several ways. First, the hot SW plasma is cooled via charge exchange. Second, and mainly as a result of this cooling effect, the HP becomes smaller and the overall flow structure simpler, see the comparison in Fig. 2. The MD, TD, and RS may vanish for certain parameters, resulting in a completely subsonic HS. Moreover, the tailward sonic line may close at finite distance from the Sun. Third, the heliotail cross section is more circular compared to MHD runs, indicating a tendency of ideal MHD to overestimate the deforming effect of the ISMF pressure (see also Pogorelov et al. 2008). Using a similar approach, Wood et al. (2014) found the directional deflection of the heliotail axis induced by the ISMF to be relatively small (probably no more than $10^{\circ}$), in consistency with Lyman-alpha absorption of stellar light. Fourth, charge-exchanging neutrals were found to induce a Rayleigh-Taylor-like instability at the heliospheric flanks by mimicking an effective gravity force, with the effect becoming more pronounced as the number of included neutral fluids is increased (Borovikov et al. 2008). As a tangential discontinuity, the HP is typically found to be prone to the Kelvin-Helmholtz instability in pure hydro simulations (in which the occasionally observed stability may occur as an artifact of low resolution). The stabilizing effect of a flow-parallel magnetic field (Chandrasekhar 1961) was confirmed by Borovikov et al. (2008) in axially symmetric, high-resolution simulations, though it should be noted that, since no such stabilizing effect can be expected for the perpendicular field component, the relevance of this finding for more realistic settings is less clear. In their 3D simulations of the HP instability, Borovikov and Pogorelov (2014) argue that it can be strongly enhanced during the periods of lower magnetic field on the heliospheric side of the HP, which are inevitable over solar cycles. The opposite is not true: the HP instability is not increasing in the absence of ISMF.

Finally, a vital ingredient to realistic large-scale heliospheric models is the fact that the SW plasma is turbulent on a multitude of scales (e.g. Bruno et al. 2005), and that this fact impacts many aspects of the heliosphere’s properties, most notably those pertaining to the energy transfer in and heating of the SW, as well as the scattering of energetic particles (Li et al. 2003). However, since most of these smaller scales cannot be resolved by classic grid-based approaches, a viable recourse is the use of Reynolds averaging, whereby a quantity $Q \in \{\mathbf{V}, \mathbf{B}, \rho \}$ is split into a “large-scale” part $\langle Q\rangle$ and a fluctuation $q \equiv Q - \langle Q\rangle$, with $\langle \cdot \rangle$ denoting a spatial averaging operator. This leads to coupled equations for the both the large-scale “background” fields and for quantities based on their small-scale contributions, such as the turbulent cross helicity density $\langle \mathbf{u} \cdot \mathbf{b}/\sqrt{\rho} \rangle$ and (twice) the total energy $\langle u^{2} + b^{2}/\rho \rangle$ per unit mass, which require phenomenological closure. Such models exist for the radial SW in 1D (e.g. Matthaeus et al. 1994; Zank et al. 1996) and 3D (e.g. Usmanov et al. 2014; Wiengarten et al. 2016), and have more recently been extended to include solar cycle effects (Adhikari et al. 2014) and then self-consistently to the 3D global heliosphere (Usmanov et al. 2016). More details can be found in the review by Fraternale et al. (2022).

After this general introduction to the field of numerical modeling of the large-scale heliosphere, the next three sections describe three such models developed by three different groups.

## The Models Implemented in the Multi-Scale Fluid-Kinetic Simulation Suite

To simulate properties of the SW, which is collisionless with respect to Coulomb collisions, it is necessary to identify a set of boundary conditions for plasma properties and the magnetic field vector. We lack measurements to specify such conditions beyond a so-called critical sphere, where the radial velocity component exceeds the fast magnetosonic speed. Remote observations of the solar magnetic field are made routinely in the photosphere, with instruments such as the Solar and heliospheric Observer (SOHO) Helioseismic and Magnetic Imager (MDI), the National Solar Observatory Global Oscillation Network Group (GONG), and the Solar Dynamics Observatory (SDO) Helioseismic and Magnetic Imager (HMI). These data can be used as boundary conditions for solar coronal models which propagate the SW beyond the critical sphere to 1 au, where the results can then be used as boundary conditions for simulations in the outer heliosphere. The *Parker Solar Probe* (*PSP*) mission (Kasper et al. 2019) is aimed to measure kinetic properties of the SW plasma to heliocentric distances below $10~R_{\odot}$, and is expected to answer the fundamental questions related to SW acceleration and transport.

**Fig. 3:**
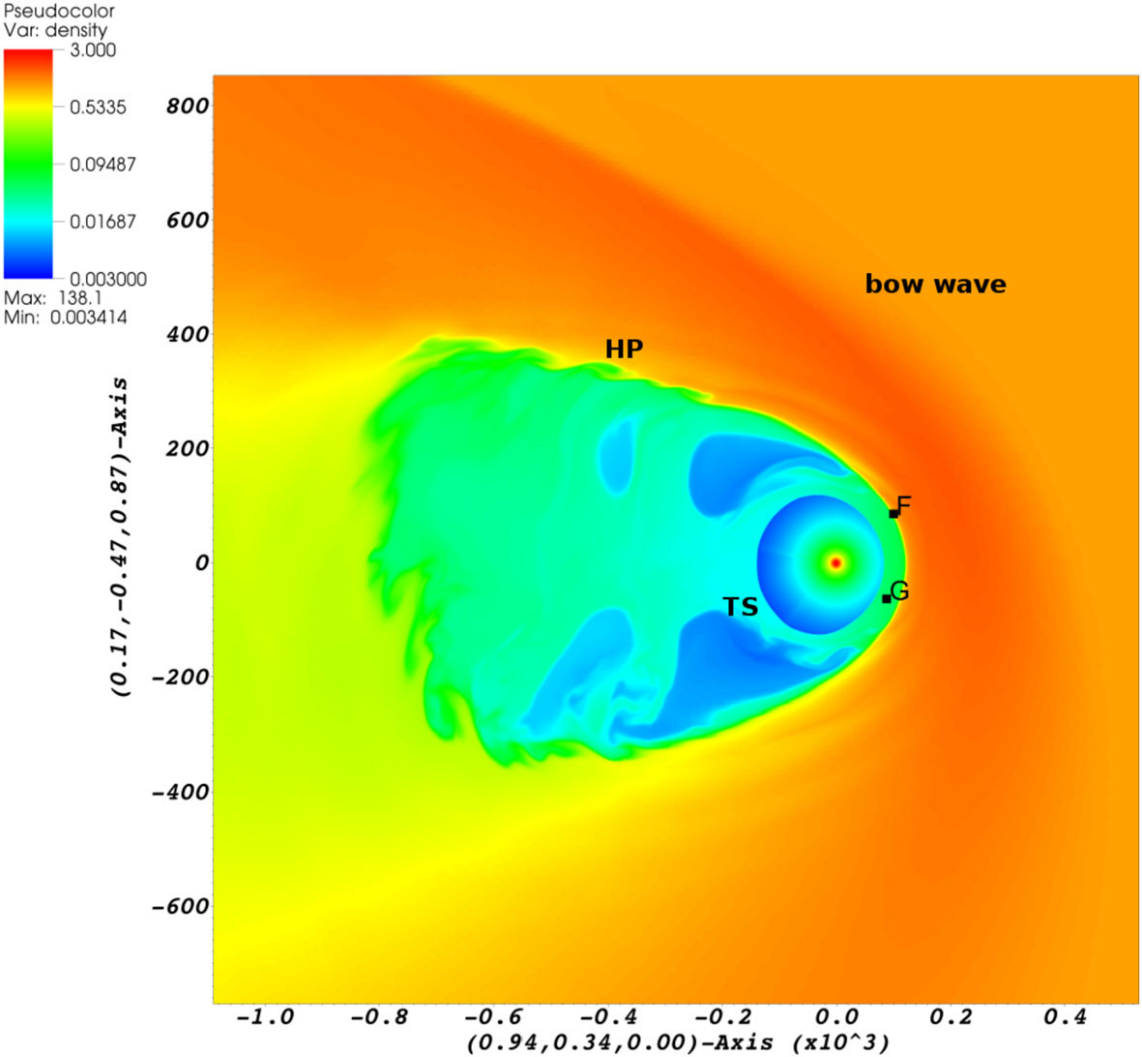
The picture of the SW–LISM interaction is shown through the plasma density distribution in the plane formed by the *V1* and *V2* trajectories (Pogorelov et al. 2015). Letters $F$ and $G$ show the spacecraft positions in 2015. While the HP crossing distance at *V1* is closely reproduced, it is also predicted that *V2* may cross the HP at a similar distance

The SW–LISM interaction is influenced by the neutral particles to a considerable extent (Wallis 1971, 1975; Gruntman 1982). As new populations of neutral atoms are born in the SW and LISM, some of these can propagate far upstream into the LISM and modify both the TS and the HP (see Fig. 3), both observed in situ by the *V1* and *V2* spacecraft (Stone et al. 2005, 2008, 2013; Gurnett et al. 2015).

The LISM plasma is collisional on scales of about 0.3–4 au (Fraternale and Pogorelov 2021), but is only partially ionized. Charge exchange between ions and atoms plays a major role in the SW–LISM interaction to such extent that the existence of a BS cannot be confirmed knowing the properties of the unperturbed LISM only (Pogorelov et al. 2017b). In addition, nonthermal pickup ions (PUIs) are created (Möbius et al. 1985; Gloeckler et al. 2009), which generate turbulence heating up the thermal ions. The heliosphere beyond the ionization cavity is dominated thermally by PUIs (Burlaga et al. 1994; Richardson et al. 1995; Zank 1999; Zank et al. 2014), which are of importance also in the VLISM. Both the charge exchange and PUI transport phenomena require kinetic treatment. PUIs are measured in situ by the *Ulysses* and *New Horizons* (*NH*) spacecraft (McComas et al. 2017b).

Charge exchange of PUIs with neutral atoms creates secondary, energetic neutral atoms (ENAs), which can propagate to near-Earth distances from their birth locations beyond the TS. The fluxes of ENAs were measured in the past by *SOHO* (Hilchenbach et al. 1998) and *Cassini* (Krimigis et al. 2009), and have been measured by the *Interstellar Boundary Explorer* (*IBEX*) since 2009 (McComas et al. 2017a). Since the ENA properties bear imprints of the parent PUIs, it is possible to deconvolve 3D properties of the heliosphere and LISM from ENA measurements (Gruntman et al. 2001; Heerikhuisen et al. 2010, 2014; Zirnstein et al. 2016; McComas et al. 2018b). Crossing of collisionless shocks by a non-Maxwellian plasma is a fundamental, unresolved problem of plasma physics (Gedalin et al. 2020, 2021a,b). In situ observations help develop the theory of this phenomenon. Moreover, a lot of observational data can be explained satisfactorily only on the basis of time-dependent, data-driven models involving the combination of MHD and kinetic scales.

The heliospheric and SW–LISM interaction models implemented in the Multi-Scale Fluid-Kinetic Simulation Suite (MS-FLUKSS) are aimed at obtaining a quantitative understanding of the dynamical heliosphere, from its solar origin to its interaction with the LISM, thus providing the heliospheric community with a data-driven suite of models of the Sun-to-LISM connection. The heliospheric model describes the relevant physical processes and helps interpreting spacecraft observations of turbulent plasma in the SW and LISM. It involves a coronal model, which in turn is driven by measured solar magnetic fields. MS-FLUKSS makes it possible to investigate physical phenomena affecting the measured ENA fluxes and their evolution in time for all observed energy ranges. MS-FLUKSS modeling accounts for substantially non-Maxwellian distributions of neutral atoms and plasma in the presence of discontinuities, turbulence, and ion acceleration effects. Validated by observational data, theoretical and modeling results obtained with MS-FLUKSS link kinetic and fluid physical scales, help interpret those observations, and build a framework for the interpretation of future *IMAP* observations (McComas et al. 2018a).

### Coupling the Inner Heliosphere with the LISM

The coronal models typically provide us with the time-dependent boundary conditions on a sphere of about $20\text{--}25~R_{\odot}$. The physical processes beyond this “critical” surface are somewhat simpler than those in the solar corona and therefore can be modeled with (Reynolds-averaged) MHD equations accompanied, wherever necessary, with additional equations describing the transport of neutral atoms (kinetic or multi-fluid), their charge exchange with ions, PUI production and transport, and turbulence/wave-particle interaction (Usmanov and Goldstein 2006; Usmanov et al. 2012; Zank 2015, 2016; Pogorelov et al. 2009d, 2017a; Izmodenov 2018).

It is not computationally efficient to perform SW–LISM interaction simulations in the computational region that starts at 0.1 au and extends to thousands of au. For this reason, we initially obtain solutions for $0.1< r<10~\text{au}$ (the inner heliosphere region). This (inner-heliospheric) simulation stage follows the coronal modeling stage. Although charge exchange becomes especially efficient between 5 and 10 au, some neutral atoms of LISM origin do penetrate to distances of 1 au and closer, where they give birth to PUIs, the properties of which are measured, e.g., by *ACE* and *Ulysses* (Zhang et al. 2019). These measurements, as well as *NH* data, are used for validation of our numerical models (Kim et al. 2017). Some of these results are presented in Fraternale et al. (2022). The final, outer heliospheric, stage connects the SW flow at 10 au to distances far into the LISM. All physical quantities obtained at the outer boundary of the previous stage are used as the inner boundary conditions for the next stage. These are saved to HDF5^2^ files, which include positions in space and time at which the data are saved. To implement this three-stage approach, MS-FLUKSS has an option to be run with the time-dependent inner boundary conditions saved in such files. This approach includes the possibility of re-interpolation of data both in space and time between stages.

Our physical model for the plasma flow assumes that charged and neutral particles are governed by different sets of equations (MHD, gas dynamic, and kinetic) self-consistently connected by the source terms responsible for ionization-recombination and charge exchange between these particles. Such terms have their components in the hydrodynamic part of the MHD system for the ion mixture – mass, momentum, and energy conservation equations:

18 $$\begin{aligned} H^{\mathrm{d}} =& m_{\mathrm{p}}n_{\mathrm{H}}\nu _{\mathrm{ph}},\quad n_{ \mathrm{H}}=\int f_{\mathrm{H}}(\mathbf{v}_{\mathrm{H}}) \, \mathrm{d}\mathbf{v}_{\mathrm{H}}, \end{aligned}$$

19 $$\begin{aligned} \mathbf{H}^{\mathrm{m}} =& \int m_{\mathrm{p}}\nu _{\mathrm{ph}} \mathbf{v}_{\mathrm{H}}f_{\mathrm{H}}(\mathbf{v}_{\mathrm{H}}) \, \mathrm{d}\mathbf{v}_{\mathrm{H}}+ \end{aligned}$$

20 $$\begin{aligned} & {} +\iint m_{\mathrm{p}}v_{\mathrm{rel}} \sigma _{\mathrm{cx}} (v_{\mathrm{rel}})(\mathbf{v}_{\mathrm{H}}-\mathbf{v})f_{\mathrm{H}}( \mathbf{v}_{\mathrm{H}}) \sum _{i\in \{\mathrm{p},\mathrm{PUI}\}} f_{i}( \mathbf{v}) \, \mathrm{d}\mathbf{v}_{\mathrm{H}}\, \mathrm{d} \mathbf{v}, \\ H^{\mathrm{e}} =& n_{\mathrm{H}}\nu _{\mathrm{ph}} E_{\mathrm{ph}}+H_{\mathrm{turb}} +\frac{m_{\mathrm{p}}}{2} \left [\int \nu _{\mathrm{ph}} \mathbf{v}^{2}_{\mathrm{H}}f_{\mathrm{H}}(\mathbf{v}_{\mathrm{H}}) \, \mathrm{d}\mathbf{v}_{\mathrm{H}}\right . + \\ & {}+\bigg. \iint m_{\mathrm{p}}v_{\mathrm{rel}} \sigma _{\mathrm{cx}} (v_{\mathrm{rel}})(\mathbf{v}^{2}_{\mathrm{H}}-\mathbf{v}^{2})f_{ \mathrm{H}}(\mathbf{v}_{\mathrm{H}}) \sum _{i\in \{\mathrm{p}, \mathrm{PUI}\}} f_{i}(\mathbf{v}) \, \mathrm{d}\mathbf{v}_{\mathrm{H}} \, \mathrm{d}\mathbf{v} \bigg], \end{aligned}$$

where $\nu _{\mathrm{ph}}$, $E_{\mathrm{ph}}$, $\sigma _{\mathrm{cx}}$, and $v_{\mathrm{rel}}$ are the photoionization frequency, ionization energy, charge exchange cross-section, and velocity of neutrals relative to ions. The quantities being integrated are the H atom and ion velocity distribution functions, $f_{\mathrm{H}}$ and $f_{i}$, and the plasma and neutral fluid velocity vectors, $\mathbf{v}$ and $\mathbf{v}_{\mathrm{H}}$. The term $H_{\mathrm{turb}}$ describes the energy source due to turbulence generated by PUIs and may be obtained either from a turbulence model (e.g. Breech et al. 2008; Zank et al. 2012; Adhikari et al. 2019) or from the kinetic treatment of the wave-particle interaction (Gamayunov et al. 2012).

One of the capabilities implemented in MS-FLUKSS is related to tracking of surfaces that propagate passively through the computational regions. These can be the HP, the heliospheric current sheet (HCS), the boundary between the sector and non-sector magnetic field regions in the HS, etc. The tracking is performed with the level-set method (Borovikov et al. 2011). Its implementation requires that the positions of the chosen surfaces are known functions of time on the inner boundary.

**Fig. 4:**
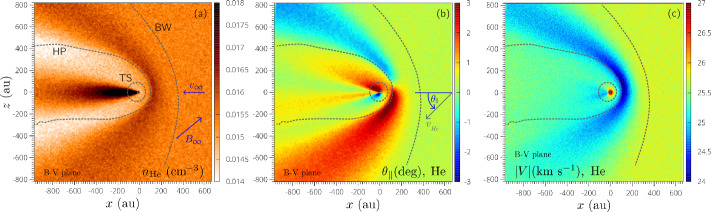
Distributions of He atom number density (panel **a**), deflection (**b**), and velocity magnitude (**c**) in the $B$–$V$ plane (in this figure, the $x$-axis is directed antiparallel to $\mathbf{V}_{\mathrm{LISM}}$, the $y$-axis is parallel to $\mathbf{V}_{\mathrm{LISM}}\times \mathbf{B}_{\mathrm{LISM}}$, the $z$-axis is therefore in the $B$–$V$ plane, pointing in the northern hemisphere). Helium and hydrogen atoms are treated kinetically. The boundary conditions used in this simulation are the same as in Zirnstein et al. (2016) (with $B_{\mathrm{LISM}}=2.93~\upmu \text{G}$), with the He parameters as in Bzowski et al. (2019). Here, the bow wave can be identified, since it reveals itself as a weak discontinuity. Adapted from Fraternale et al. (2021)

The heliospheric model and its implementations in MS-FLUKSS address the complexity of the ISMF–heliospheric magnetic field (HMF) coupling at the HP and charge exchange between neutral and charged particles (Borovikov et al. 2008, 2011, 2012; Borovikov and Pogorelov 2014; Pogorelov and Zank 2006; Pogorelov et al. 2008, 2009b,c,d, 2012, 2013b, 2015, 2016, 2017a,b; Heerikhuisen and Pogorelov 2011; Heerikhuisen et al. 2010, 2014). Adaptive mesh refinement (AMR) has been implemented into MS-FLUKSS (Kryukov et al. 2006, 2012; Pogorelov et al. 2009a). The general block scheme of MS-FLUKSS is given in Pogorelov et al. (2014), while the suite is continuously evolving with new models and features added. For example, we have recently added (Fraternale et al. 2021) the interstellar He atoms and $\text{He}^{+}$ ions to the model in a self-consistent way, which makes it possible to (i) investigate the kinetic transport of He atoms through the heliosphere towards the *IBEX* detector and (ii) derive information necessary for updating the properties of the LISM in a way appropriate for modeling the SW–LISM interaction. Figure 4 shows the density, deflection, and velocity distributions of neutral He atoms in the *B*–*V* plane.

**Fig. 5:**
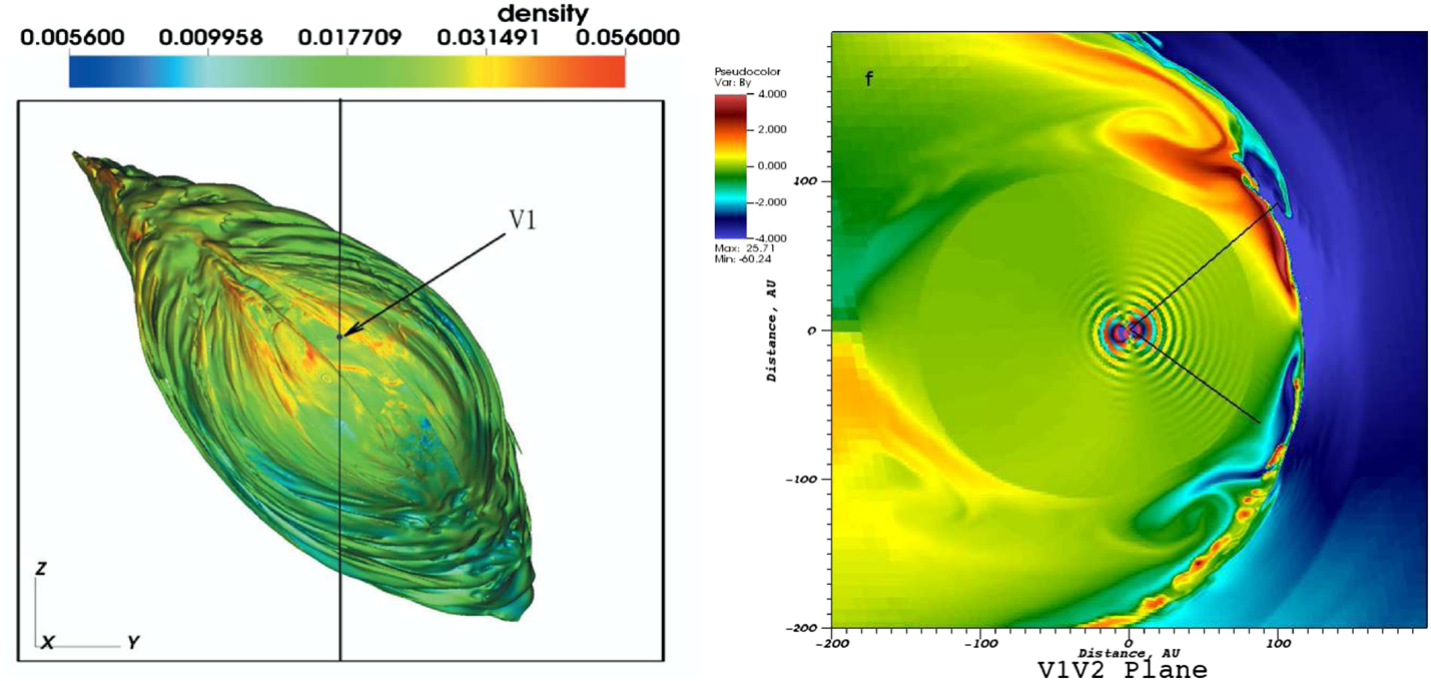
Left panel: The interstellar view of the unstable heliopause colored by plasma density values. As described by Borovikov et al. (2011), in MS-FLUKSS the HP is tracked using the level-set method (e.g., Osher and Fedkiw 2003). The global reference system used here has the $z$-axis parallel to the Sun’s spin axis, the $x$-axis belongs to the plane containing the $z$-axis and $\mathbf{V}_{\mathrm{LISM}}$ and is directed upstream into the LISM. Right panel: The sharp reversal of the $B_{y}$ magnetic field component observed in a global simulation is favorable for magnetic reconnection. Multiple instabilities are observed. Adapted from Pogorelov et al. (2017b)

In MS-FLUKSS, the transport of neutral particles throughout the heliosphere is either calculated kinetically, using a direct simulation Monte Carlo method, or with a multi-fluid approach, where neutral atoms born in thermodynamically different regions of the heliosphere are modeled with separate Euler gas dynamics systems. For data-driven problems, the application of the kinetic approach to atoms is not very efficient, so we often pursue a multi-fluid approach. Pogorelov et al. (2009d) presented a detailed comparison of plasma, neutral atom, and magnetic field distributions obtained with our 5-fluid (one plasma and four neutral fluids) and MHD-kinetic models, and revealed a good agreement between them. The HP is subject to different MHD instabilities (Florinski et al. 2005; Borovikov and Pogorelov 2014; Pogorelov et al. 2017b). Figure 5 shows the 3D topology of the HP affected by these instabilities. This solution was obtained using our multi-fluid approach and adaptive mesh refinement, to reduce the effects of numerical dissipation. Since the kinetic treatment of the neutral atom transport results in “noisy” source terms, its application to modeling instabilities may affect the outcome.

Figure 9 in Fraternale et al. (2022) shows the comparison of our simulation based on an MS-FLUKSS model with PUIs governed separately by the continuity and pressure equations, and the turbulence model of Breech et al. (2008) with *NH*, *Ulysses*, and *Voyager* observations. It is of interest that, as shown in Fig. 31 (right panel) of Fraternale et al. (2022) (see also Pogorelov et al. 2013b), when the width of sectors of positive and negative polarities is not resolved in the HS, the HMF, instead of quietly dissipating, shows features of transition to stochastic behavior, which is indicative of the effect of turbulence on the HS flow.

### Validation with Observational Data

We use in situ measurements from *NH*, *OMNI*, *PSP*, *Ulysses*, and the *Voyagers* and integral ENA fluxes at *IBEX* Zirnstein et al. (see also the paper by 2022). Capturing the time-dependent nature of the SW is crucial to understanding observations in the solar corona, heliosphere, and LISM. Coronal models can easily incorporate magnetograms obtained from different viewpoints, as may become available in the future (e.g., from *Solar Orbiter*). The coupling of the coronal and heliospheric models in MS-FLUKSS opens opportunities to reveal the fundamental physical phenomena occurring in heliosphere and LISM surrounding it.

**Fig. 6:**
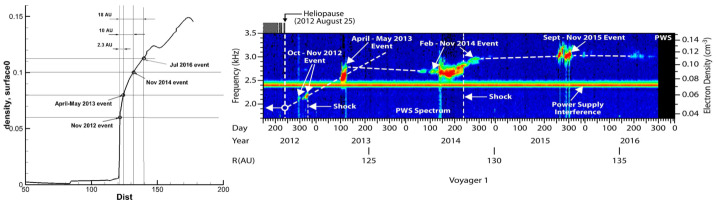
Left: Simulated plasma density distribution in the *V1* direction shows a deep heliospheric boundary layer on the LISM side of the HP. Right: A color-coded spectrogram of the wideband electric field spectral densities detected by the PWS instrument. The frequency is on the left $y$-axis, and the corresponding electron density is on the right. From Pogorelov et al. (2017b)

The SW–LISM models implemented in MS-FLUKSS have been successful in interpreting and/or predicting a number of non-trivial observations: (1) data-driven simulations of Borovikov et al. (2012), Pogorelov et al. (2009b, 2013b), Kim et al. (2017) made it possible to better understand observational data and occasionally predict them, see details in Mostafavi et al. (2022) and Fraternale et al. (2022); (2) the effect of the ISMF on the neutral hydrogen deflection plane (Lallement et al. 2005; Izmodenov et al. 2005; Pogorelov et al. 2008, 2009c); (3) strong correlation of the *IBEX* ribbon position on full-sky maps and the orientation of the $B$–$V$-plane defined by the LISM velocity and ISMF vectors, in the unperturbed LISM (Pogorelov et al. 2010; Heerikhuisen and Pogorelov 2011; Heerikhuisen et al. 2016; Zirnstein et al. 2016); (4) the modeled H density at the TS is in agreement with that derived from PUI measurements (Bzowski et al. 2009); (5) the effect of PUIs on the TS (Pogorelov et al. 2016), (6) the TS and HP positions (Pogorelov et al. 2013b, 2015; Zirnstein et al. 2016), (7) backward SW velocities at *V1* (Pogorelov et al. 2012), (8) MAG and PWS observations at *V1* on the LISM side of the HP (Pogorelov et al. 2009b, 2017b; Borovikov and Pogorelov 2014, see also Fig. 6), and (9) the observed anisotropy in the 1–10 TeV galactic cosmic ray (GCR) flux (Schwadron et al. 2014; Zhang and Pogorelov 2016; Zhang et al. 2014, 2020). The latter observation has been reproduced on the basis of our SW–LISM interaction model, which requires the heliotail to have a comet-like shape (Pogorelov et al. 2015, 2017a; Pogorelov 2016) as long as 10,000 au (see Sect. 8.2).

### Remote Sensing of the SW–LISM Interaction Using ENAs

The charge-exchange coupling between SW plasma and neutrals from the LISM creates ENAs within the heliosphere. These ENAs inherit a velocity that is a combination of the plasma bulk flow and thermal speed. As a result, ENAs born in the supersonic SW move out radially with the SW speed and escape the heliosphere, penetrating several hundred au into the LISM. This flux of ENAs from the supersonic SW is sometimes referred to as the neutral SW (Gruntman 1997). In the HS, the lower plasma flow speed and the presence of suprathermal PUIs give rise to ENAs that move in all directions, including toward the inner heliosphere, where they may be detected by spacecraft. The creation of ENAs in the SW removes energy from the plasma, which leads to a cooling of the plasma as it travels through the HS. This cooling is primarily driven by the fact that the more energetic PUIs have a higher rate of charge exchange, resulting in a source region of higher energy ENAs close to the TS.

Heliospheric ENAs are observed at a wide range of energies. Many of the ENAs from the HS have energies on the order of a few keV, and represent PUIs moderately energized by crossing the TS. Such ENAs are the primary focus of the *IBEX*-Hi instrument (Funsten et al. 2009). A subset of PUIs is more strongly energized by the TS, and can have energies of tens of keV, and have been detected by *Cassini*-INCA (Krimigis et al. 2009) and SOHO-HSTOF (Hilchenbach et al. 1998). Lower-energy neutral particles, with energies below a few hundred eV, are observed by the *IBEX*-Lo instrument (Fuselier et al. 2009). These neutrals come directly from the LISM, or through change-exchange in the VLISM. The future IMAP mission (McComas et al. 2018a) will improve on current observations by measuring neutral atom fluxes from the heliosphere over energies from tens of eV, to above 100 keV.

The data from the *IBEX* spacecraft can be presented as all-sky maps of ENA flux for specific ranges of ENA energy, or as flux as a function of energy for a particular direction in the sky. Model ENA fluxes are generally constructed through a post-processing of the SW–LISM simulation, where the contributions to the flux at 1 au are integrated along different lines of sight over the sky. The differential flux of neutral hydrogen atoms in the solar inertial frame is given by Zirnstein et al. (2013) as

21 $$ \Delta J (\mathbf {v}_{\mathrm{p}}) = \frac{1}{m_{\mathrm{H}}} f_{ \mathrm{p}}(\mathbf {v}_{\mathrm{p}}) v^{2} P(\mathbf {v}_{\mathrm{p}}) \left ( \int f_{\mathrm{H}}(\mathbf {v}_{\mathrm{H}})|\mathbf {v} - \mathbf {v}_{\mathrm{H}}| \, \sigma _{\mathrm{ex}} (|\mathbf {v} - \mathbf {v}_{\mathrm{H}}|) \, \mathrm{d}^{3} v_{\mathrm{H}}\right ) \Delta t $$

where $f$ is the velocity distribution function of each species, $P$ is the survival probability for such neutrals to reach the detection location, and $\sigma _{\mathrm{ex}}$ is the charge-exchange cross-section. We can then integrate the source of flux along lines of sight to produce simulation skymaps or ENA spectra that can be directly compared with *IBEX* data.

One of the most intensely studied features in the *IBEX* data is the so-called “ribbon” of enhanced flux that encircles the sky (McComas et al. 2009). This unexpected feature drew much speculation as to its origin (McComas et al. 2014), though early comparisons to models hinted at a connection to the draping of the LISM magnetic field around the HP (Schwadron et al. 2009). Heerikhuisen et al. (2010) implemented a model where (“primary”) ENAs exit the heliosphere, charge-exchange to become PUIs in the VLISM, and then charge-exchange again to become “secondary” ENAs (see Fig. 8). They assumed a simple model where PUIs do not scatter to full isotropy, so that as a result a signature of the magnetic field orientation in the ENA source region is imprinted on the secondary ENA flux. Subsequent analyses (Funsten et al. 2013) showed that both the observed and simulated ribbons exhibit remarkably circular geometry. The secondary ENA mechanism has become the nominal explanation (McComas et al. 2017a) of the ribbon, though various versions exist, which differ in the dynamics of PUIs in the VLISM (Schwadron and McComas 2013; Zirnstein et al. 2021).

By carefully tuning simulations to match ENA data from *IBEX*, we are able to deduce various global properties of the SW–LISM interaction. In particular, the sensitivity of the ribbon to the strength and orientation of the LISM magnetic field allows us to make remarkably precise predictions. Early results (Heerikhuisen and Pogorelov 2011) showed that the model ribbon approaches a great circle for large values of $B_{\mathrm{LISM}}$ ($\gtrsim 5~\upmu \text{G}$), but that the ribbon radius decreases systematically for weaker fields. Figure 9, from Zirnstein et al. (2016), shows a statistical analysis of a range of model heliospheres with different $B_{\mathrm{LISM}}$ vectors, for a range of *IBEX*-Hi energies. The most likely field strength is just below 3 μG. This analysis shows the best agreement between models and observations occurs for ENA energies in the 0.5–2.5 keV range (IBEX-Hi ENAs 2, 3, and 4), which corresponds to primary ENAs born with typical supersonic SW speeds of 300–750 km/s.

Another area where ENAs can help us identify the structure of the heliosphere is the heliotail. In order to compare our simulated ENAs from the heliotail with *IBEX* data, we must use a model heliosphere that includes at least a simple solar cycle such that tailward lines of sight contain regions of fast, low density, SW at high latitudes, and slower, more dense, SW at equatorial latitudes. Once the cycle has propagated through the heliosphere, we can collect ENA fluxes using Equation (21), but we must correctly account for the ENA travel time to the detector from the source region whose properties are changing in time. Figure 10 shows comparisons between *IBEX* data and the corresponding simulation results for skymaps that are centered on the downwind direction of the heliosphere. The distribution of relative flux intensity across the sky strongly suggests that the heliosphere has a heliotail very similar to that obtained in the simulation.

Finally, since the ENAs seen by *IBEX* come mostly from PUIs in the HS, IBEX spectral properties can be used to help deduce the characteristics of PUIs and their energization at the TS. While it is not feasible to trace the dynamics of PUIs on an individual level, we can make use of the conservation laws in the MHD model to estimate the total energy in the plasma-PUI mixture, since the charge-exchange source terms inject the pressure of newly formed PUIs into the MHD system. We can then define separate populations of PUIs based on how they were energized at the TS. A simple approach (used in Zank et al. 2010; Heerikhuisen et al. 2019) is to define a population (the majority) of PUIs which are energized as they are transmitted through the TS, along with a population of PUIs that reflect off the cross-shock potential and experience more significant energization. Another approach has been proposed recently by Gedalin et al. (2021b), which is based on the incorporation of the results from kinetic (test-particle and/or full particle-in cell) modeling of the TS crossing into global models. These two PUI populations can then be tracked through the HS, along with the relatively cooler core SW and a population of newly formed PUIs that are injected into the plasma as it advects away from the TS (Zirnstein et al. 2014). Such a PUI model can then be used to compute ENA flux in post-processing. For example, Shrestha et al. (2020) generated an all-sky map of the reduced $\chi ^{2}$ values between the ENA flux observed by *IBEX* and the corresponding flux computed using a simulation of the SW–LISM interaction with multiple populations of PUIs. Figure 11 shows where the model agrees well with the data, and where it does not. Not surprisingly, the ribbon, the heliotail, and polar regions do not match well since this particular simulation uses uniform slow SW and no ribbon model was included.

## Moscow 3D Kinetic-MHD Model of the Global Heliosphere

In this section we give a brief overview of the kinetic-MHD model developed by the Moscow group. The latest version of the model is described in Izmodenov and Alexashov (2015). Some new results and comparison with *Voyager* data are given in Izmodenov and Alexashov (2020). The early development of the model goes back to the pioneering paper by Baranov and Malama (1993), who developed the self-consistent kinetic-gasdynamic model for the first time. About 30 years after the publication of this paper, it has become clear that the chosen physical and numerical approaches were quite optimal. The modern model of Moscow group is based on the same hybrid kinetic-MHD approaches.

### Physical Assumptions

The main approaches and assumptions made in the model can be briefly summarized as the following:

1. Since the local ISM is partially ionized the model has two components – neutral, consisting of atomic hydrogen, and charged particles – electrons, protons (including pickup protons), ions of interstellar helium, and alpha particles in the solar wind. The dynamic role of interstellar helium ions and SW alpha particles has been explored by Izmodenov et al. (2003). Since then these components have been taken into account in the global models of the Moscow group.
  Other components have small cosmic abundances and do not have a dynamic effect on the global heliosphere. The distribution of these components can be calculated in non self-consistent manner, as it has been done for atomic and ionized interstellar oxygen (Izmodenov et al. 1999a, 2004), for atomic and ionized interstellar helium (e.g. Kubiak et al. 2014), and for interstellar dust (e.g. Alexashov et al. 2016; Mishchenko et al. 2020; Godenko and Izmodenov 2021). Distributions of pickup protons and heliospheric ENAs can, in principle, be calculated in a non-self-consistent approach that is quite appropriate to compare with data (Baliukin et al. 2022b). However, as it has been shown by Malama et al. (2006), separate kinetic treatment of pickup component results in redistribution of energy throughout the heliosheath and effects on the global structure of the flow including positions of the TS and HP. Such effects are lost in the kinematic approach.
2. The two components – neutrals and plasma – interact each with other. The main physical process of this interaction is resonant charge exchange (H atoms with protons), although the processes of photoionization and ionization of H atoms by electron impact can be important in some regions of the heliosphere (for example, in the heliosheath or in the supersonic solar wind). The significant effect of resonant charge exchange is connected with the large cross section of such collision, which is a function of the relative velocity of colliding particles.
3. The interstellar neutral component should be calculated in the frame of the kinetic approach because the mean free path of the hydrogen atoms (with respect to charge exchange) is comparable with the size of the heliosphere (see, e.g. Izmodenov et al. 2000). The multi-fluid approach that is often employed in alternative models does not have a physically established background. Nevertheless, it has been shown by Alexashov and Izmodenov (2005) (for one set of model parameters and in 2D) that a multi-fluid model may produce plasma distributions that are quite close to the distributions obtained in the frame of kinetic-gasdynamic models. However, there is no guarantee that the kinetic and multi-fluid results are close enough for arbitrary parameters. There is no way to quantify the level of uncertainty introduced by multi-fluid approximations.
4. All charged particles (of both solar and interstellar origin) are considered as a single-fluid, ideal, perfect, mono-atomic gas. The fluid approach is valid for interstellar plasma because this medium is collisional. Indeed, the mean free path of interstellar protons with respect of Coulomb collisions is about or less than 1 au (e.g., Baranov and Ruderman 2013; Fraternale et al. 2021). At the same time the SW is a collisionless plasma and strictly speaking the fluid approach is not very well justified. However, there is a common belief (supported by many observations) that the collisionless plasma behaves as a fluid and ‘maxwellization’ of the distribution function appears due to wave-particle interaction.
  It is important to note that the single-fluid approach for all charged components is based on two major assumptions. The first one is that all components are co-moving. This assumption is valid for pickup protons when the magnetic field is frozen into the solar wind/interstellar plasma. In this case the newly created (pickup) protons are picked up by the heliospheric electromagnetic field, so all components move together with the same bulk velocity. The second assumption is that the velocity distribution function of pickup protons becomes isotropic (in the bulk plasma rest frame) very quickly (as compared with the characteristic time of convection). With these assumptions, single-fluid equations remain valid. However, the right parts of these equations should include the distribution function for pickup protons (see, for example, Equations (18)–(20)).
  Furthermore, one should either calculate the distribution function for pickup protons by solving corresponding kinetic equation for pickup protons, as it was done in the papers by Malama et al. (2006), Chalov et al. (2016), and more recently by Baliukin et al. (2020) and Baliukin et al. (2022b), or make another assumption of Maxwellian distribution for the mixture of thermal and pickup protons. Such an assumption has been made in all so-called single-fluid plasma models, including the Baranov and Malama (1993) model, and its further developments with the exemption of the paper mentioned above.
5. Due to the charge change the fluid equations for the plasma component and the kinetic equation for the neutral component are coupled. The right sides of the fluid plasma equations contain source terms which are integrals of the velocity distribution function of the H atom component. Also, the collision term in the kinetic equation for H atoms depends on the plasma gasdynamic parameters. Therefore, the fluid equations for plasma and the kinetic equation for neutrals need to be solved self-consistently. This makes the problem quite complex.
6. In the considered mathematical model the magnetic fields are treated in a non-dissipating approach. This means that the magnetic diffusion and Hall terms are neglected in the equation for the magnetic field, and the system of ideal MHD equations are solved. Such a theoretical approach is also employed in the models of other groups (for instance, by the BU group, see Sect. 7). However, contrary to the other groups we extend this ‘ideal’ approach (as far as possible) in physical formulation into the numerical approach that will be described below. For example, our numerical method does not allow for numerical reconnection at the heliopause or in the heliospheric current sheet.
7. Another complexity in the modeling of the global heliosphere is its time-dependent nature. On timescales of hundreds of years the main driver for time-dependence is the variations of the SW parameters and, in particular, the dynamic pressure. The most pronounced periodic variations appear with the 11-year solar cycle. The Moscow model allows to have time-dependent solutions with one important assumption, namely that the solutions should be periodic. The period can be chosen rather arbitrary. For example, 66-year periodic solution has been considered by Izmodenov et al. (2005). However, the obtained period for the entire solution was the same as the period imposed by the boundary conditions, i.e. 11 years. Izmodenov et al. (2008) have considered the model with a realistic solar cycle when the OMNIWeb and Wind data for 22 years (from years 1984.5 to 2006.5) have been employed in the boundary conditions.
  In the more modern 3D kinetic-MHD model, the variations of the solar wind with time and heliolatitude has been taken into account. Namely, three sets of experimental data are used:
  - In the ecliptic plane we use data (solar wind density and speed) from the OMNI 2 database. The OMNI 2 data set contains hourly resolution solar wind magnetic field and plasma data from many spacecraft in geocentric orbit and in orbit about the $\text{L}_{1}$ Lagrange point.
  - Heliolatitudinal variations of SW speed are taken from analysis of the interplanetary scintillation (IPS) data. The results of Sokół et al. (2013) for one-year average latitudinal profiles of the SW speed with a resolution of $10^{\circ}$ has been used. Data are available from 1990 to 2011.
  - Heliolatitudinal variations of SW mass flux are derived from the analysis of SOHO/SWAN full sky maps of the backscattered Lyman-alpha intensities (Quémerais et al. 2006; Lallement et al. 2010; Katushkina et al. 2013). Inversion procedures Quémerais et al. (see 2006, for details) allow to obtain SW mass flux as a function of time and heliolatitude with a temporal resolution of approximately one day and angular resolution of $10^{\circ}$. Data are available from 1996 to 2011.
8. The dynamic effects of GCRs on the global heliosphere was studied (e.g. Myasnikov et al. 2000b,a; Alexashov et al. 2004) with the simplified approach when the diffusive equation for effective pressure of the cosmic rays has been solved together with the Euler equations for the plasma component. The latter equations have source terms that are proportional to the gradient of the cosmic-ray pressure. Myasnikov et al. (2000b) have found in the frame of two-component model (plasma plus GCRs) that GCRs could considerably modify the shape and structure of the TS and the BS, and change the heliocentric distances to the HP and the BS. For the three-component self-consistent model (plasma + H atoms + GCRs) it has been shown by Myasnikov et al. (2000a) that the GCR influence on the plasma flow is negligible when compared with the influence of H atoms. The exception is the BS, a structure which can be modified by cosmic rays. Therefore, it can be concluded for the heliosphere that GCRs do not have a significant dynamic influence on the global heliosphere, and can therefore be treated kinematically.
  The dynamical influence of anomalous cosmic rays (ACRs) has been studied by Alexashov et al. (2004). The paper provides a parametric study by varying the diffusion coefficient because it is poorly known in the outer heliosphere and especially in the HS. It has been shown that the effect of ACRs leads to the formation of a smooth precursor, followed by the subshock, and to a shift of the subshock towards larger distances in the upwind direction. The intensity of the subshock and the magnitude of the shift depend on the value of the diffusion coefficient with the largest shift (about 4 au) occurring at medium values of the diffusion coefficient. The postshock temperature of the SW plasma is lower in the case of a cosmic-ray-modified TS compared to a shock without ACRs. The decrease in temperature results in a decrease in the number density of hydrogen atoms originating in the region between the TS and the HP. The cosmic-ray pressure downstream of the TS is comparable with the thermal plasma pressure for small values of the diffusion coefficient when the diffusive length scale is much smaller than the distance to the shock. An upwind-downwind asymmetry in the cosmic-ray energy distribution due to difference in the amount of energy injected in ACRs in the up- and downwind parts of the TS has also been obtained.
9. It is important to note that the work of the Moscow group has been mainly focused on the obtaining and analyzing stationary or periodic solutions. The study of instabilities was less explored until recently. This is connected with the fact that analytical studies are extremely difficult (see, however, Baranov et al. 1992; Chalov 2019) and nearly impossible at the nonlinear stage, while in the numerical studies of instabilities it is very difficult to distinguish between real physical instabilities and numerical instabilities induced by the numerical scheme. For example, it is possible to demonstrate that small (and theoretically justified) changes in the classical Godunov scheme applying in the numerical cells near the heliopause may switch on/off the heliopause instability. Nevertheless, a numerical study of the Kelvin-Helmholtz instability of the heliopause has recently been done also by Korolkov et al. (2020).

### Numerical Approaches

In order to obtain a self-consistent steady-state or periodic solution of the system of kinetic and ideal MHD equations, the global iteration method proposed by Baranov and Malama (1993) is used. The non-stationary versions of MHD equations are solved using a 3D modification of the finite-volume Godunov-type scheme that employs a Harten-Lax-van Leer Discontinuity (HLLD) MHD Riemann solver Miyoshi and Kusano (see, for example 2005). A Chakravarthy-Osher TVD limiter with the possibility to choose compression parameters (Chakravarthy and Osher 1983) is used to increase the resolution properties of the first-order accuracy scheme. The solver has been adopted to a 3D moving grid with the possibility to fit discontinuities. The so-called soft fitting technique (Godunov et al. 1979; Myasnikov 1997) has been employed. The chosen numerical scheme allows us to fit all of the major discontinuities – the HP, the TS, and the BS, when the latter exists. In particular, the HP is unambiguously identified by ensuring that during its motion, this surface is always tangential to both magnetic fieldlines and velocity streamlines, and that the pressure balance is satisfied. All of the other discontinuities (if any) can be captured by the scheme as well.

A steady-state or periodic solutions of the MHD equations are obtained using the time-relaxation method. In other words, we solve the non-stationary equations with stationary or periodic boundary conditions for long periods of time when, for the stationary case, the temporal derivatives of all of the parameters become negligibly small and the flow pattern reaches steady state in the computational domain, or, for the periodic case, periodic variations of plasma parameters are established. It is checked that the obtained steady-state/periodic solutions persist over very long periods of time, such that possible instabilities do not destroy the numerical solution.

To satisfy the condition of $\nabla \cdot \mathbf{B}=0$ we follow the well-known procedure suggested by Powell et al. (1999), which consists of adding to the right hand side parts of the non-stationary versions of the MHD equations those terms which are proportional to $\nabla \cdot \mathbf{B}$, namely $-\mathbf{B}(\nabla \cdot \mathbf{B})/(4\pi )$ to the momentum equation, $-(\mathbf{V}\cdot \mathbf{B})(\nabla \cdot \mathbf{B})/(4\pi )$ to the energy equation, and $-\mathbf{V}(\nabla \cdot \mathbf{B})$ to the magnetic field induction equation.

The results presented in most of the publications use a specific non-regular moving grid that allows us to perform exact fitting of the TS and HP. This grid also allows us to decrease the sizes of the cells (thereby increasing the number of cells) in the vicinity of these discontinuities. The example of a typical numerical grid in the $ZX$ and $XY$ planes is shown in Fig. 12. Note that the numerical codes allow to perform calculations on both non-moving orthogonal (rectangular) adaptive grids (see Korolkov and Izmodenov 2021; Titova et al. 2021) as well as on the non-regular grids with different geometry of computational cells including tetrahedral and Dirichlet’s cells. Ideal gasdynamic testing shows that all grids produce the same results, including the structure in the tail region with the Mach disk, reflected shock, and secondary tangential discontinuity.

The question of the outlet boundary conditions in the tail is also important. In the case of pure gasdynamics the SW outflow is subsonic in the tail. This requires to set the value of the Riemann invariants for the incoming (in the computational domain) characteristic. Fortunately, this problem does not appear in the self-consistent kinetic-gasdynamic problem because it has been shown by Izmodenov and Alexashov (2003) that the outlet plasma flow is supersonic in this case due to charge exchange with interstellar protons.

The convergence of the numerical solution has been verified by changing the grid resolution up to two times in all directions and by changing cell sizes specifically in the vicinity of the discontinuities. Also, the numerical solution has been tested on the extended grids where the size of the computational domain was increased up to two times. In particular, the tail region varied in these calculations from 1000 au up to 5500 au.

The kinetic equation for the H-atom component has been solved using a Monte Carlo method with spatial and physical splitting of the particle trajectories. The employed method is very similar (although not fully identical) to the one described in Malama (1991). Monte Carlo calculations have been performed using the same computational grid as described for the plasma component. No additional interpolation is employed.

The main idea of the geometrical splitting consists in the following. All space is divided into $N$ zones, which are spherical layers in our case. The initial velocity distribution function $f$ is split into the sum of several functions $f_{i}$ according to $f = \sum _{i=1}^{N} \mu _{i} {f_{i}}$, where $f_{i}$ is chosen in such a way that particles from this function must enter (during their motion within the heliosphere) into zone $i$. The splitting of trajectories allows to increase statistics of Monte Carlo calculations by a factor of $10^{5...6}$.

### Application of the Moscow Model to Analyze of Spacecraft Data

The results obtained using the framework of the Moscow model have been used in the analyses of various observations. The most pronounced one, perhaps, is the prediction of the hydrogen wall around the HP. This wall was firstly reported by Baranov et al. (1991) and then discovered in Lyman-alpha absorption spectra toward $\upalpha $ Cen by Linsky and Wood (1996). Later the Moscow model was applied to analyses of Lyman-alpha absorption towards nearby stars (Izmodenov et al. 1999b; Wood et al. 2007b,a, 2009, 2014). Another important application of the Moscow model is for analyses of backscattered solar Lyman-alpha data (e.g. Baliukin et al. 2022a, and references therein). An important achievement of the model that we can mention is its ability to quantitatively explain the deflection of the interstellar H atom flow vector in the heliosphere (Izmodenov et al. 2005; Katushkina et al. 2015a) discovered by Lallement et al. (2005, 2010). The Moscow model has also been applied to analyses of backscattered solar Lyman-alpha observations made by SOHO, HST, Voyagers, and Pioneer 10 (e.g. Katushkina et al. 2019, 2017, 2016, 2015b, 2013; Vincent et al. 2014; Quémerais et al. 2008; Pryor et al. 2008; Gangopadhyay et al. 2006). Furthermore, the Moscow model has been employed in the results presented in the papers of Sokół et al. (2022), Galli et al. (2022), Herbst (2022), and Baliukin et al. (2022a) from this journal.

Examples of the results obtained with the modern version of the Moscow model are shown in Fig. 13. Panels A and B clearly illustrate the stretching and pushing of the HP under the action of the interstellar field. The HP has a blunt shape in *V1* direction and an oblong shape in *V2* direction. Therefore, $B_{R} >0$ and $B_{R} <0$ in the respective directions of *V1* and *V2*. This case is in agreement with *Voyager* observations. Since the model is time-dependent through the solar cycle, the fluctuations of the TS and the HP are shown in Panels C and D. The TS fluctuates by $\sim12$ to 15 au from minimal to maximal distance depending in the direction that is in agreement with previous time-dependent models (Izmodenov et al. 2005, 2008). The HP distances are $\sim123.5~\text{au}$ in August 2012 for the *V1* direction and $\sim121.5~\text{au}$ in November 2018 for the *V2* direction. These distances do not exactly coincide with the distances of actual HP crossings by the *Voyagers*, but are still quite close. The model results show that the HP moves inwards from 2009 to 2016 and then outwards. This is surely connected with the minimum of the SW dynamic pressure from 2007/2008 to 2015.

The model vs. data comparison shows that just beyond at the HP all three components of the IMF obtained in the model match those measured by both *Voyagers* (Panels E and F). Data for *V1* are restricted by the closest vicinity of the crossing, while *V1* magnetometer data after the HP crossing are publicly available from 2012 until the end of 2017. It is seen that the $B_{N}$ and $B_{T}$ components match the data for the entire period of available observations, although tiny details of the component fluctuations are different in model and data. Significant differences between model results and data exist for the $B_{R}$ component. In the Model, $B_{R}$ remains nearly constant for the entire period from 2012 to 2017 and further, while in the data, it is nearly constant for 2013–2014 and then gradually decreases. Two bumps on the generally decreasing slope are associated with disturbances induced by merged interaction regions (Burlaga et al. 2018, 2019). The model-data difference in $B_{R}$ along the *V1* trajectory may be connected with the time-fluctuations. Indeed, when the heliopause moves in and out it acts as a piston. Moving in it compresses the magnetic field lines. Therefore, the tangential component of the interstellar magnetic field increases. The radial motion of the heliopause should increase the $B_{R}$ component too because the direction of the magnetic field should be parallel to the heliopause surface. The fact that fluctuations of all magnetic field components correlate is clearly seen in both *V1* data and in the model results (Panel A). However, the level of fluctuations in the model is somewhat smaller than in the data. This may be connected, with the dataset for the solar wind parameters that has been employed in the model. For example, it may be due to 27 day time averaging that has been performed for the solar wind parameters in the model. Averaging, of course, reduces the level of fluctuation. In addition, some instabilities may occur at the heliopause which are not considered in the model.

## The BU Model

There are several MHD models in the community currently. In this section we cover the Boston University (BU) model. One important aspect is that MHD models make several assumptions in solving the equations. In order to understand the solutions and the comparison with observations, it is important to take into account the assumptions each model makes. The BU group chose to simplify the solutions and each factor included in the model to understand the main physical drivers of the heliosphere that is still not well understood. Several outstanding questions relating to the heliosphere are present. One of them is the shape of the heliosphere that is being actively debated (see Sect. 8). The BU model also allows for time-dependent solar wind conditions (described below), although in several of our works we chose a simplified boundary with uniform solar wind representative of solar maximum, to explore the main physical drivers with the numerical models.

The BU model uses the *Space Weather Modeling Framework (SWMF)*, which is a modeling tool that is able to evaluate different regions of the heliosphere at varying scales. Within the SWMF is the OH component, which is based on the *Block-Adaptive Tree Solar wind Roe-Type Upwind Scheme (BATS-R-US)* solver (Tóth et al. 2012), which is a 3D, block adaptive, upwind finite-volume MHD code that is highly parallel. Opher et al. (2003) adapted BATS-R-US to the outer heliosphere as the OH component, which is a global 3D multi-fluid simulation. Within SWMF, multiple options are present for the treatment of the solar wind plasma and neutrals. The solar wind plasma can either be treated as a single-ion fluid consisting of thermal solar wind ions and PUIs (Opher et al. 2015) or as multiple ion fluids with the MHD equations treating thermal solar wind ions and PUIs separately (Opher et al. 2020). For the neutrals, they can either be treated in a multi-fluid fashion as four separate neutral populations characterized by the region in which they are created (Opher et al. 2009, 2015) or kinetically by solving the Boltzmann equation via a Direct Simulation Monte Carlo Method (Tenishev et al. 2021; Michael et al. 2021).

As a stand-alone component within SWMF, the OH component is capable of treating multiple ion fluids in addition to multiple neutral fluids (Opher et al. 2020). In this multi-fluid treatment of the heliosphere, the ideal MHD equations are solved for the ion species, while Euler’s equations are solved separately for the individual neutral H populations, with each neutral population corresponding to a different region of the heliosphere. Source terms from McNutt et al. (1998) connect the ion and neutral fluids for a single-ion plasma approximation, which treats the thermal solar wind ion and pickup ions (PUIs) as a single fluid population.

Neutrals streaming from the ISM should be treated kinetically. In its current form, the OH component allows for the solution of the heliosphere to be obtained through different modules. The ionized component can be treated as a single or multiple ion species. Although not accurate as a kinetic treatment, a multi-fluid treatment (four different fluids for neutrals for the different regions in the heliosphere where they are created, see Opher et al. 2009) is numerically faster. Ultimately the BU model will implement separate ions for SW and PUIs while treating the neutrals kinetically. By doing the latter, the PT component provides an alternative treatment of neutrals to the multi-fluid treatment which exists in the OH component. The PT component of SWMF is based on the Adaptive Mesh Particle Simulation (AMPS) and is able to treat neutral atoms kinetically. AMPS is a global, 3D kinetic particle code which solves the Boltzmann equation using a Direct Simulation Monte Carlo method (Tenishev et al. 2021), with the initial purpose to solve the Boltzmann equation for a dusty, partially ionized, multi-species gas in cometary comae. The PT component is able to act as a standalone code, or be coupled to other components, such as OH in this scenario. The BU model couples the PT component to the OH component, and in doing so the PT component is used to solve the Boltzmann equation for neutral H atoms streaming through the domain and only incorporates effects due to charge exchange. The neutral H atoms are the only modeled neutral population and are injected at the outer boundary with a Maxwell-Boltzmann distribution.

All of the current BU models treat the solar magnetic field as unipolar in both hemispheres. This is based on the work of Opher et al. (2015), and the unipolar treatment of the solar magnetic field is used to eliminate spurious numerical effects due to numerical diffusion and reconnection of the solar magnetic field across the heliospheric current sheet (Michael et al. 2018). Using a unipolar magnetic field configuration leads to reconnection occurring on the port side of the heliosphere (the left side of the heliosphere as it moves through the ISM as seen when looking from the Sun outwards) and in the heliotail for the BU model. Using a dipolar magnetic field configuration induces spurious numerical reconnection across the heliospheric current sheet and between the solar magnetic field and the ISMF at the heliopause, which leads to a draped ISMF that differs from observations (Michael et al. 2018). To determine the location of the HP in the BU model, the discontinuity is captured after the MHD solution is obtained. Based on the solar wind velocity streamlines at the nose and flanks of the heliosphere, as well as non-reconnected solar magnetic field lines, we fit MHD variables that best capture these lines. The isosurface is determined by specifying a value for a particular MHD variable that is shown to have all solar wind velocity streamlines and non-reconnected solar magnetic field lines within the heliopause, and ISM velocity streamlines and reconnected field lines draping around the HP.

Based on kinetic studies, (Opher et al. 2017) argued that reconnection is suppressed at the nose while occurring at the flanks. The use of a unipolar solar magnetic field is able to suppress reconnection at the nose while allowing it at the flanks, and is able to explain *V1* and *V2* magnetic field data ahead of the heliopause (Opher et al. 2017, 2020).

The BU model is able to model time-dependent phenomena by varying the SW speed, density (Provornikova et al. 2014), and magnetic field intensity (Michael et al. 2015). The polarity of the solar magnetic field is not varied to avoid large regions of numerical dissipation within the HS that are unrealistic (Michael et al. 2018). Variations in SW speed are determined by interplanetary scintillation (IPS) data (Sokół et al. 2013, 2015) for variations in latitude at 1 au and by using in situ data from the OMNI database in the ecliptic plane. SOHO/SWAN Lyman-alpha intensity maps are used to determine the heliolatitudinal distribution of the interstellar hydrogen ionization rate, which can be used to obtain the charge-exchange rate in the solar wind. Coupled with the IPS-derived SW speed, the charge-exchange rate can yield the SW density at 1 au. The solar magnetic field intensity is included by fitting 27-day averages of the field magnitude average of the magnetic field strength at 1 au recorded by OMNI. We assume a Parker spiral, solar wind conditions being constant in time, and a magnetic field modulated by the polar angle in the Parker solution.

The different versions of the BU model have all led to notable scientific advances. Opher et al. (2006, 2009) quantified the influence of the interstellar magnetic field in the asymmetry of the solar system (first suggested by Izmodenov et al. 2005) as seen by several *Voyager* observations. This asymmetry is found not only in north-south (Opher et al. 2006, 2009) but also east-west direction (Opher et al. 2007). Opher et al. (2015) were able to show that the solar magnetic field, although dynamically has a pressure much smaller than the thermal pressure (the plasma beta in the HS is $\gg1$), is a critical factor in shaping the heliosphere structure and shape. A more detailed discussion on this topic is included in Sect. 8.1.

Kornbleuth et al. (2021b) compared the heliospheric solutions of the BU model and the Moscow model, which suppresses magnetic reconnection at the heliopause, and showed that regardless of heliopause treatment both models show a confinement of the solar wind plasma by the solar magnetic field (Fig. 14). This is demonstrated through the mass flux, which is organized by the solar magnetic field in the heliotail. The comparison also showed that the twisting of the azimuthal magnetic field at the heliopause, which occurs during reconnection, leads to an increase in magnetic pressure outside of the heliosphere and a compression of the heliosphere. Opher et al. (2020) explored the effect of PUIs on the heliosphere by including the PUIs as a separate fluid population. They found that the depletion of PUIs, which were originally created in the supersonic solar wind, via charge exchange led to the cooling of the heliosphere and a smaller, rounder heliospheric shape than is seen when thermal solar wind ions and PUIs are treated as one population as is often done in the community for simplicity. The PUIs charge exchange and leave the system acting as a source of energy sink in the system (see Fig. 15). Another development from the BU model is the demonstration of a Rayleigh-Taylor instability in the HS (Opher et al. 2020). The charge exchange between the HS plasma and streaming interstellar neutrals results in an effective gravity that causes an instability to form (see Fig. 16) along the axis of the solar magnetic field. The instability leads to the opening of the heliotail as it causes the lobes of the heliotail at high latitudes to erode.

## The Split-Tail vs. Comet-Tail Debate

Since the early days of Parker and his contemporaries, the geometric shape of the global downwind heliosphere has been pictured as a single, long, roughly cylindrical tube with approximately elliptical cross section. Only more recently has an alternative possibility been suggested, namely that of two distinct jets which emanate from the polar regions, then are bent into the downwind direction through interaction with the interstellar flow (thereby forming a semi-toroidal shape that has been dubbed the “croissant heliosphere”), and finally dissolve relatively quickly due to the onset of turbulence. At this point, the community has not reached a consensus on whether the actual shape of the heliosphere is more appropriately described by these “split-tail,” or the more traditional “comet-tail” models. To properly reflect the state of this debate, arguments in support of the former are summarized in Sect. 8.1 by M. Opher and M. Kornbleuth. N. Pogorelov, F. Fraternale, and J. Heerikhuisen argue for the latter in Sect. 8.2. V.V. Izmodenov offers his comments on the situation and the state of the controversy in Sect. 8.3.

### Arguments in Support of Split-Tail Models

The issue of the shape of the heliosphere is being actively debated. It was shown in the work of Opher et al. (2015) and in the subsequent work of Drake et al. (2015) that the solar magnetic field tension plays a critical role funneling the solar wind in the heliosheath into two high-latitude lobes. Recently it was settled that among different models, as BU, and Moscow Kornbleuth et al. (2021b) (both with kinetic treatment of neutrals and same latitudinal solar wind) the heliotail plasma was confined by the solar magnetic field. The difference resides in that in the BU model at 400 au downtail the ISM flows in between the two confined jets while in the Moscow model the two jets are embedded in a long comet-like tail. Moreover, as shown by Kornbleuth et al. (2021a) and discussed by Dialynas et al. (2022), ENA maps at *IBEX-Hi* cannot distinguish between the two solutions. We expect that higher-energy ($>10~\text{keV}$) ENA maps will differ between these models, since PUIs survive at these energies for longer distances before being depleted due to charge exchange, so ENA data at these higher energy channels could help determine the structure of the heliotail.

The main difference between the two models is the ISM that flows between the two collimated jets in the HS. The main question is why this happens. Recently there was an important discovery that shed light on the topic: the realization that a Rayleigh-Taylor-like instability may occur along the solar magnetic axis in the heliosphere (Fig. 16). This instability destroys the coherence of the heliospheric jets and generates turbulence which leads to magnetic reconnection, allowing LISM material to enter the heliotail (Opher et al. 2021). There is still the issue to understand the exact path from the instability becoming highly non-linear to allowing reconnection to proceed and the ISM to penetrate between the two lobes. The Rayleigh-Taylor-like instability induces turbulence in fluid scales of several au. The other question is why this instability does not appear in the Moscow model that presents a steady-state laminar solution. The indication is that this has to do with the main difference between the two models on treating the HP boundary, one that has boundaries that use a “fitting” techniques (Moscow) while the other allows for communication across the boundary (BU). The other pressing question is how this instability develops under different conditions (introduced by e.g., the solar cycle).

Another important conclusion recently arrived at (Opher et al. 2021) is that the kink instability is not the driving mechanism to the instability of the heliospheric jets (as argued in Sect. 8.2 and speculated in Pogorelov et al. 2015), and that the heliospheric jets are in fact stable to the kink instability. Kink or sausage instabilities in jets are stabilized if there is a sufficiently strong axial field. Previous works studied the stability of an azimuthal magnetic field (Parker 1974; Roberts 1956), concluding that kink or sausage instabilities were at play when the axial field was weak. In particular, previous works studying the heliospheric tail (Yu 1974) ascribe the turbulence seen in simulations (Opher et al. 2015; Pogorelov et al. 2015) to kink instabilities. This was also the explanation offered by Opher et al. (2015) based on studies like Begelman (1998) of cases of weak axial fields in astrophysical jets. However, it is also known that shear flows can stabilize the kink modes (e.g. Shumlak and Hartman 1995). We show that the magnetic field structure of the heliospheric jets has a speed shear that stabilizes the jets for kink or sausage instabilities.

The original work (Opher et al. 2015) to argue that the shape of the heliosphere is “croissant-like” was done with a multi-fluid treatment of neutral H and with uniform solar wind. In this model, the solar magnetic field confines the solar wind plasma into two northward/southward columns that develop into lobes in the heliotail. The ISM is able to flow between these lobes, where mixing of the interstellar and solar wind plasma occurs along reconnected magnetic field lines (Opher et al. 2017). It was then revisited (Opher et al. 2020) including pickup ions as a separate fluid. There have been suggestions that different solar wind conditions and the treatment of neutral H would remove the two-jet structure of the heliotail and result in a long, comet-like tail. Pogorelov et al. (2015) suggested that the inclusion of a dipolar solar magnetic field configuration (as opposed to the unipolar configuration used by Opher et al. 2015) would lead to a long tail. A dipolar magnetic field configuration leads to a large amount of magnetic field dissipation and introduces numerical artifacts into models since magnetic reconnection numerically is captured at a grid cell sizes while the physical mechanism is taking place sub-grid (on kinetic scales). Michael et al. (2018) investigated the effects of unipolar and dipolar magnetic field configurations of the SW in the BU model and found that the two-jet structure (or “croissant-like”) shape of the heliotail persists because the flat current sheet present in the dipolar model is insufficient to fully erode the magnetic tension force.

A kinetic neutral treatment, which was also not present in the work of Opher et al. (2015), has been suggested (Pogorelov et al. 2015) as a mechanism for removing the two jets. Izmodenov and Alexashov (2015) demonstrated that despite the collimation of the solar wind in the heliosheath obtained in the frame of 3D kinetic-MHD Moscow model the heliopause has traditional open-tail shape. They also suggested that momentum transfer due to charge exchange at low latitudes pushes the heliopause further from the Sun when a kinetic neutral treatment is used. Michael et al. (2021) and Kornbleuth et al. (2021b) used a kinetic neutral treatment with uniform and latitudinally-varying solar wind conditions, respectively, in the BU model and both works found that a “croissant-like” shape persists even in the presence of a kinetic neutral treatment.

We agree therefore with the discussion in Sect. 8.3 that sub-grid kinetic effects such reconnection should be included in models – this is particularly challenging in the heliosphere where the kinetic scales (km) are separated from the fluid scales (au) by orders of magnitude. The BU model chooses to suppress reconnection as the Moscow model in regions where there are no indications that reconnection is taking place while allowing it (port side) to occur where there are indications that this is the case (Opher et al. 2017). While the studies proposed in Sect. 8.3 are interesting, Opher et al. (2021) indicate that the newly found Rayleigh-Taylor instability along the axis of the solar magnetic jets is responsible for the ISM flowing between the jets – so effort should be done to understand why models such as Moscow do not present such instabilities, as well as understanding the evolution and impact of such instability on the global structure of the heliosphere and on acceleration of particles.

The “croissant-model” has not yet been put to the test to see if it is consistent with TeV GCRs or with Lyman-alpha measurements towards nearby stars. There are critical challenges though on these two indirect probes. TeV GCRs are in particular sensitive to magnetic polarities. Down the heliotail several solar cycles are present and the issue of how to incorporate solar cycles that include a flipping of the solar magnetic field polarity without introducing large spurious regions of magnetic dissipation (e.g. Michael et al. 2018) is a challenge. Lyman-alpha observations towards the tail are another potentially great tool to probe the structure of the heliotail but it requires careful inclusion of the distribution of neutral atoms in the HS.

We disagree with the claims voiced in Sect. 8.2 that the split-tail heliosphere is inconsistent with observations. As shown by Kornbleuth et al. (2021b), the shape of both the split-tail and comet-tail models agree at the nose. Kornbleuth et al. (2021a) showed that the *IBEX* ENAs can only see the nose shape of both BU and Moscow model that agree within *IBEX* observations, with the cooling length presenting limitations on the ability of *IBEX* to distinguish the heliotail’s shape. Both the split-tail and comet-tail models show an organization of the heliosheath plasma by the solar magnetic field (Kornbleuth et al. 2021b). Opher et al. (2021) showed agreement of the split-tail model with both *V1* and *V2* data across the HP and argue that this has to do with reconnection occurring in the flanks, and as such a comet-tail model is not required for observational agreement. Lastly, one cannot say that the split-tail model disagrees with TeV observations given that the split-tail model has not yet been compared with TeV observations. Moreover, Kornbleuth et al. (2021b) has shown that different physical mechanisms in different models can lead to changes between solutions, so the work of Zhang and Pogorelov (2016) can only be used to argue that the comet-tail model agrees with observations, but cannot make claims on the comparison with the split-tail model.

The future direction is for a model to include all components of the heliosphere – thermal and suprathermal components of the solar wind, PUIs, ACRs, GCRs, as well as kinetic neutral atoms. In addition, the model should define, quantify, and simulate all relevant processes that effect these components, from microphysical processes such as reconnection and turbulence to PUI and cosmic-ray transport and acceleration. This is part of the SHIELD^3^ project.

### Arguments in Support of a Comet-Like Heliotail and Against Unipolar Heliospheric Magnetic Field

In contrast to Parker (1961), Yu (1974) proposed an alternative model of the heliosphere where the heliotail splits into two branches. This model has recently been revived in the modeling and interpretation efforts by Opher et al. (2015), Pogorelov et al. (2015) and Pogorelov (2016). The heliotail splitting in Yu’s scenario is due to the SW collimation by the spiral HMF. While the presence of above-mentioned collimation cannot be excluded under certain circumstances, as shown by Yu (1974) and Pogorelov et al. (2015), it is ultimately destroyed by “kinking” when the SW temperature drops below a certain threshold. Moreover, the collimation itself does not necessarily result in the tail splitting, particularly, because it strongly depends on the assumption of unipolar heliospheric magnetic field (Opher et al. 2015; Pogorelov et al. 2015). Interestingly, there seems to be no splitting even in the assumption of a flat heliospheric current sheet (HCS), which is true when the Sun’s magnetic and rotation axes coincide (Pogorelov et al. 2015). Pogorelov et al. (2017b, 2021) demonstrated that this approach may increase the HMF pressure in the HS up to 50 times as compared with *V1* and *V2* measurements. Figure 17 (left panel) shows a comparison of magnetic field measurements performed by *V2* with the steady simulation of Pogorelov et al. (2021), which qualitatively agrees with Opher et al. (2020), where the observational data are misrepresented. In particular, it was stated that magnetic field measurements performed by V2 (actually also by V1) belong to the rectangles similar to the one which was taken from Opher et al. (2020) and inserted into the left panel of Fig. 17. In reality, they do not, neither ahead of the HP, nor after it. This makes solutions based on the assumption of unipolar magnetic field deficient. This conclusion does not depend on the particular choice of a code or a numerical method. On the other hand, the numerical simulations of Pogorelov et al. (2013a), where the SW variations at the inner boundary are governed by *Ulysses* data, show that the average magnetic field behavior is reproduced much better in the HCS presence, even if the latter is not resolved (see Fig. 17, right panel). It is of importance to realize that artificial magnetic reconnections should be expected regardless of whether the HMF is data-driven or assumed unipolar. This is because of the uncertainty in the choice of the HMF direction, which changes to the opposite every 11 years. The HMF sector structure cannot be expected to be resolved in any simulation with a fixed grid resolution. Hopefully, the issue might be addressed if the effects of turbulence are taken into account explicitly.

*Voyager* data show that magnetic field pressure in the HS plays a minor role. The assumption of unipolar magnetic field changes the situation to the opposite, and an artificially increased magnetic pressure starts to affect the flow. Strikingly, the SW collimation of the type proposed by Yu (1974), is impossible in the presence of solar cycle effects, even if the HMF is assumed entirely unipolar (see the discussion in Pogorelov et al. 2017a). This is caused by the variation of the latitudinal extent of the slow SW, where the plasma density is considerably higher than in the fast SW.

**Fig. 7:**
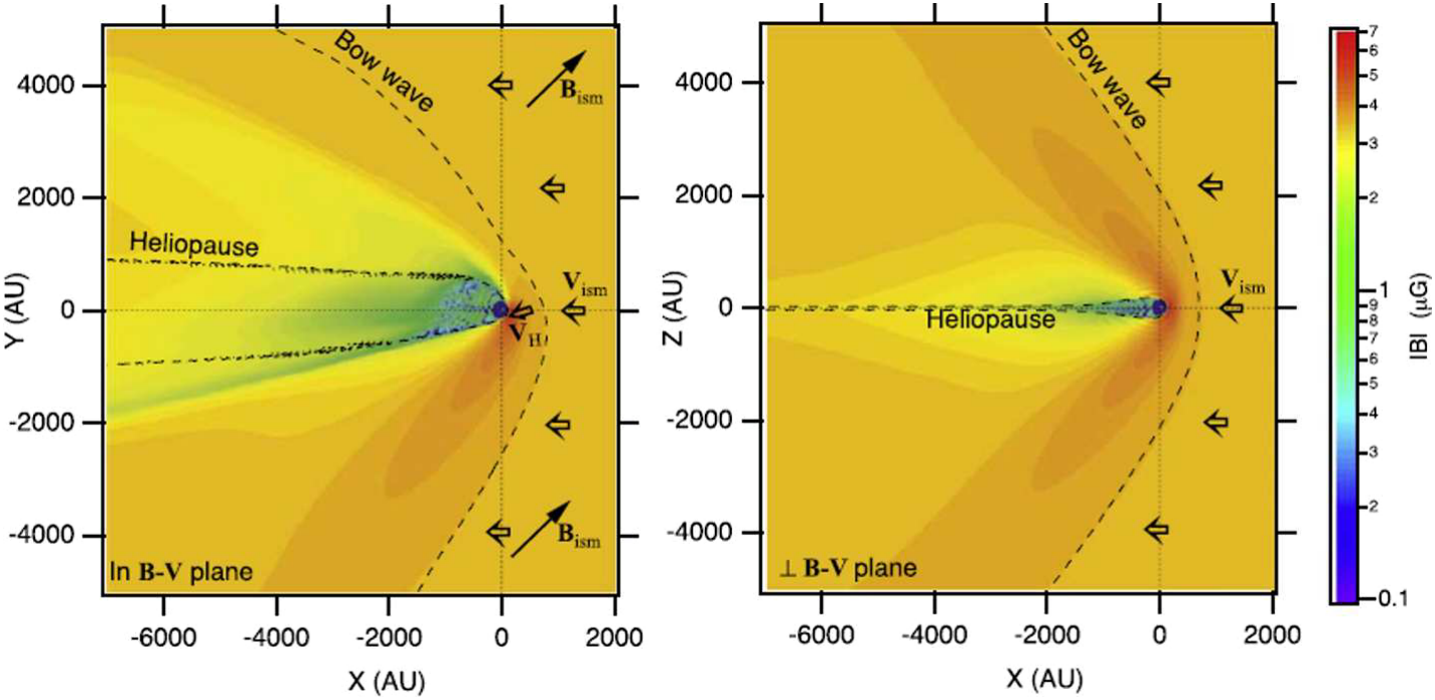
The distributions of magnetic field strength in the $B$–$V$ plane (left panel) and in the plane perpendicular to the $B$–$V$ plane (right panel). In this figure, the $x$-axis is antiparallel to $\mathbf{V}_{\mathrm{ISM}}$, the $z$-axis is parallel to $\mathbf{B}_{\mathrm{ISM}}\times \mathbf{V}_{\mathrm{ISM}}$, the $y$-axis in the $B$–$V$ plane. The dashed lines indicate the positions of the HP and the heliospheric bow wave. The deviation of the magnetic field and plasma flow from their values in the unperturbed LISM distorts the picture of GCR anisotropy. The arrows show the direction of the ISMF and LISM velocity vectors, $\mathbf{B}_{\mathrm{ISM}}$ and $\mathbf{V}_{\mathrm{ISM}}$, and the velocity vector, $\mathbf{V}_{\mathrm{H}}$, of the deflected flow of neutral hydrogen entering the heliosphere. The heliotail is truncated at a distance of 2,500 au from the right-hand boundary. Reproduced from Zhang et al. (2020)

Our short-tail heliosphere disagrees with the observed anisotropy of 1–10 TeV GCRs (Zhang and Pogorelov 2016; Zhang et al. 2020), which requires a heliotail exceeding 10,000 au (see Fig. 7). It does not account for the presence of a jump in magnetic field magnitude across the heliopause accompanied by absence of rotation of the magnetic field vector observed by both *V1* and *V2*, while data-driven models do (Pogorelov et al. 2021). It is also inconsistent with the shape of the heliosphere derived from *IBEX* observations (Reisenfeld et al. 2021). Korolkov and Izmodenov (2021) showed that the heliosphere can acquire a tube-like (another description of the croissant) shape similar to those in Yu (1974) and Opher et al. (2015) only for fast magnetosonic Mach numbers lower than 0.3 in the unperturbed LISM, which is not applicable to the heliosphere. It is known, however, that the observed astrotails can have remarkably different shapes (Chatterjee and Cordes 2002; Sahai and Chronopoulos 2010).

**Fig. 8:**
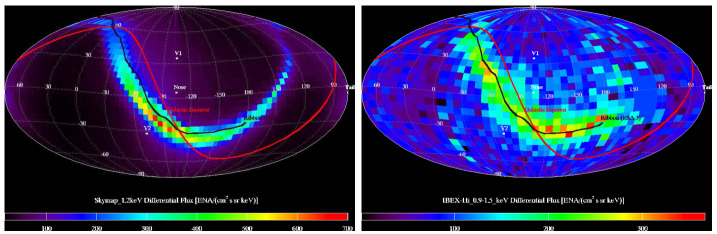
The first all-sky map from a simulation of the *IBEX* ribbon (left), along with the corresponding data from *IBEX* (right). Taken from Heerikhuisen et al. (2010)

**Fig. 9:**
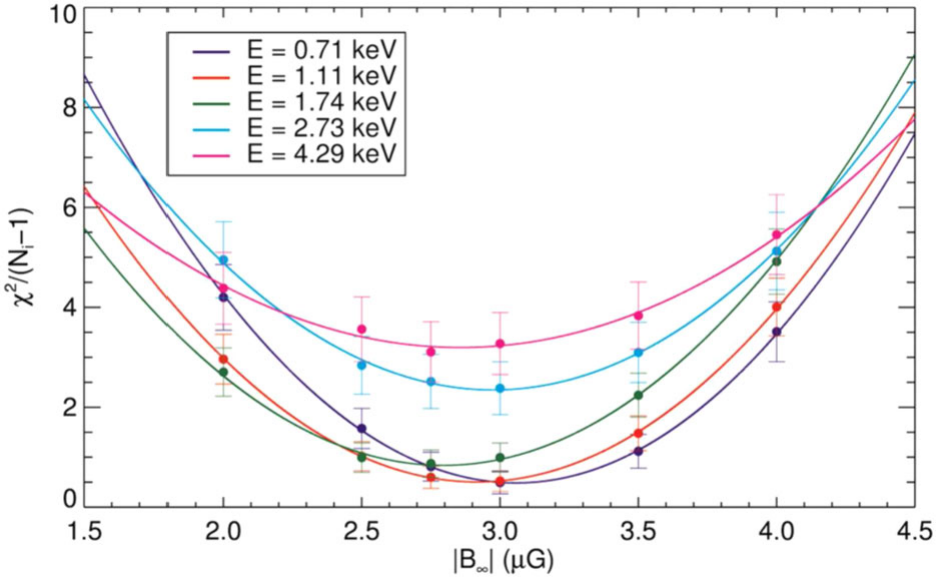
Results of the $\chi ^{2}$ minimization process for determining the most likely magnetic field strength in the unperturbed LISM (from Zirnstein et al. 2016). Here we compare the radius of the simulated ribbon with the value determined using *IBEX* observations

**Fig. 10:**
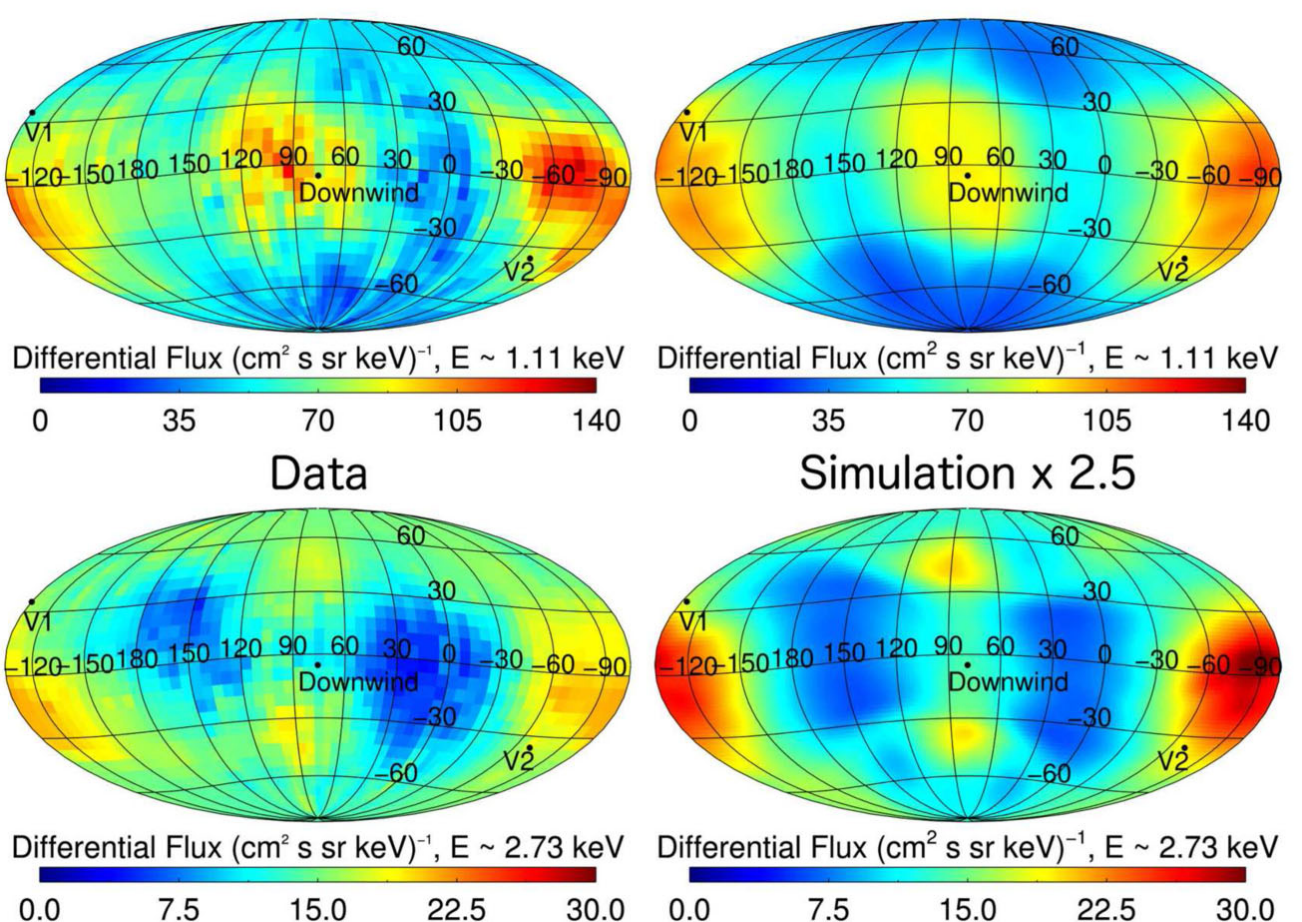
Comparison of ENA maps from *IBEX* (left, with the ribbon removed) and the map of simulated ENA flux due to charge exchange in the HS (from Zirnstein et al. 2017). At lower energies (1.1 keV, top row) the tail flux is concentrated in a $60^{\circ}\times 60^{\circ}$ region in the downtail direction. At higher energies signatures of the stream of HS plasma that was fast SW appear at latitudes above $\sim 60$ degrees. Note that the simulated flux has been multiplied by 2.5, indicating that while distribution of relative flux intensity agrees well with the data, some physics may still be missing from the model

**Fig. 11:**
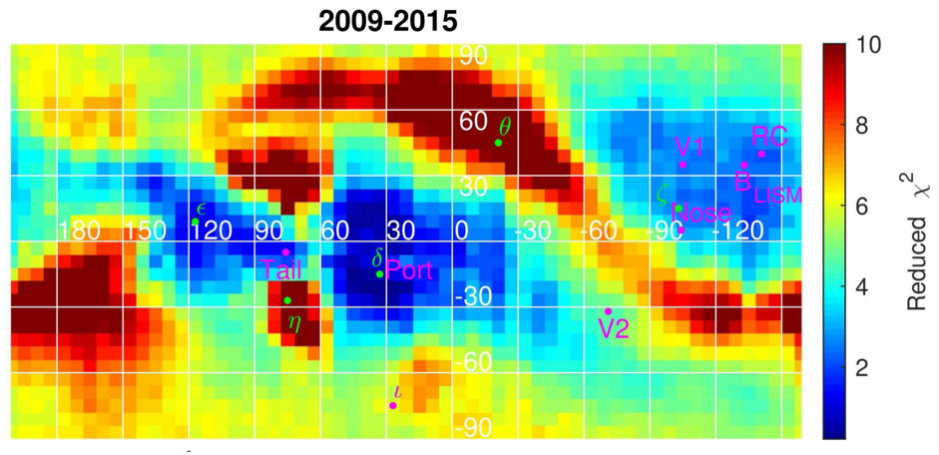
All-sky map of the reduced $\chi ^{2}$ between model ENA flux and *IBEX* data (from Shrestha et al. 2020). For each direction we have summed over fluxes corresponding the IBEX-Hi ESA 2 to 6. The two bright spots seen toward the heliotail in Fig. 10 show up here as poor fits (high $\chi ^{2}$) since this simulation does not use time-dependent SW. Similarly the ribbon is a poor fit, since the simulation did not include a ribbon model

**Fig. 12:**
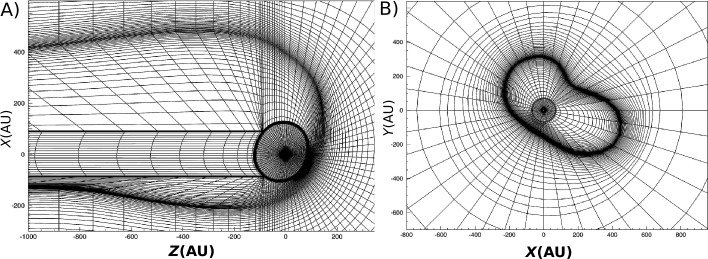
Demonstration of the computational grid in the $ZX$ plane (panel **A**) and in the plane parallel to the $XY$ plane at $z=-500~\text{au}$ (panel **B**). The $Z$ axis is directed toward the interstellar flow, the $X$ axis is perpendicular to $Z$ and in the $B$–$V$-plane such $(\mathbf{B}_{\mathrm{ism}})_{x} < 0$, and the $Y$ axis completes the right-handed system of coordinates. Adapted from Izmodenov and Alexashov (2015)

In summary, solutions to the same system of MHD equations from the different models are still qualitatively, and in most of cases quantitatively, similar for the same sets of boundary conditions. However, the choice of boundary conditions determines those solutions. To reproduce and interpret spacecraft observations, the boundary conditions should be data-driven. The assumption of unipolar heliospheric magnetic fields without the heliospheric current sheet is not only non-physical. As shown above, it clearly creates artifacts, which require explanation. It is worth noting from this perspective that not only the observational data, but also simulation results always have explanations. As a result, the artificial solutions obtained with questionable assumptions can also be explained. This creates a closed, never-ending circle of result-explanation sequences.

The complexity of the problem is that there are no direct observations in the heliotail. On the other hand, no existing indirect measurement seems to require the heliosphere to acquire a croissant shape (Zirnstein et al. 2018; Heerikhuisen et al. 2019; Reisenfeld et al. 2021). This includes the Lyman-alpha absorption profiles in the directions towards nearby stars (Zank et al. 2013; Wood et al. 2014), which are in fact consistent with a long heliotail pointing into the downwind direction. Czechowski et al. (2020) have recently shown that ENA data obtained using the standard (comet-like) model of the heliosphere are close in flux magnitude to observations by *IBEX*, *SOHO*-HSTOF, and partly those by *Cassini*-INCA (except for the 5.2–13.5 keV energy channel) in the energy range 3–88 keV. It was found that the ENA flux from the tail dominates at high energy (in agreement with HSTOF, but not INCA). At low energy, the “comet-like” model produces ENA fluxes of similar strength from the upwind and downwind directions, which was earlier used as an argument for a bubble-like heliosphere.

We may never know the exact structure of the heliotail on the basis of observational data. An expectation of a global physical model that would describe all time and length scales affecting the SW–LISM interaction is counterproductive. It suffices to say that current ideal MHD models have been successful in the interpretation of important observational data. Many of them are described in this paper.

### Pro and Contra Arguments Based on Moscow Model Results

As it is clearly seen from the two previous subsections, the current numerical kinetic-MHD models of the global heliosphere produce very different qualitative results for the shape of the heliopause by solving the same set of mathematical equations with the same (or very similar) sets of boundary conditions. In principle, such a situation is not acceptable and discredits the numerical models in the eyes of heliospheric community. It is highly necessary to understand the reason of the difference. Possible physical and observational arguments for tube-like (croissant) and comet shapes of the heliopause were laid out in previous subsections. However, it is important to underline that numerically we solve a mathematical problem that is determined by the set of equations and the boundary conditions. All physics is determined by the equations. Since the ideal MHD equations do not take into account viscous or magnetic dissipation, the argumentation that these processes influence the global structure is beyond the mathematical problem solved. It is quite apparent that the reason of difference between models is to be found in the numerical approaches. Direct detailed comparison of the numerical BU and Moscow models have been done recently (Kornbleuth et al. 2021a). They found similarities and differences in the solutions but did not answer the main question which result is correct and why. It is quite apparent for us that numerical dissipation (and, in particular, reconnection) should be reduced as much as possible. The consideration of uni-polar heliospheric field and ensuring the absence of reconnection at the heliopause are the ways to reduce effects of numerical dissipation. In order to account for reconnection one should modify the mathematical model rather than rely on numerical effects.

In our point of view, there is quite straightforward way to explore the global shape of the heliopause mathematically being in the frame of ideal MHD approach. We suggest and started to realize the working program that should allow to establish in details how the global structure of the heliosphere bifurcates from a tube-like to a comet-like shape. This, we believe, will allow to establish the ground truth of the numerical models. To do it we suggest to decompose the complex kinetic-MHD problem into simpler steps and perform parametric studies at each step by exploring how the bifurcation from tube-like shape to comet-shape appears.

Since the physical driver of the solar wind collimation toward the poles is the azimuthal magnetic field we started with the model of the magnetized stellar wind outflows into the resting interstellar gas. Such problem has been studied by Golikov et al. (2017) for different sets of dimensionless parameters. As expected after passing the TS the stellar wind collimates into two jets directed towards axis of the stellar rotations. This forms a tube-like heliopause. The width of the tube depends on the magnitude of the stellar magnetic field and the value of interstellar gas pressure.

The next step has been done recently by Korolkov and Izmodenov (2021). The interaction of the magnetized stellar wind with the interstellar non-magnetized flow has been explored. For simplicity the interstellar magnetic field and neutral component have been neglected. With these simplifications the solution of the problem depends only on two dimensionless parameters: the gas-dynamical Mach number of the interstellar flow, $M_{\infty}$, and the Alfvén Mach number of the stellar wind, $M_{A}$. For small values of $M_{\infty}$, the structure of the flow has a tube-like structure similar to the one by Golikov et al. (2017). However, the shape of the tube is twisted toward the tail. The flow pattern is quite complex in the tail and includes the reverse interstellar flow and the stagnation point in the interstellar gas. By increasing the interstellar Mach number, it was found that for any given $M_{A}$ there is a critical value of $M_{\mathrm{crit}}$ at which the flow pattern changes its structure from tube-like to comet-like tail. For $M_{\infty}> M_{\mathrm{crit}}$ the heliopause has an open structure in the tail. Therefore, the heliospheric flow pattern bifurcates from tube-like shape to comet-like shape at $M_{\infty}= M_{\mathrm{crit}}$. For a value of $M_{\mathrm{A}} = 12$ that is close to the solar value the bifurcation appears at $M_{\infty}\approx 0.3$. Since the case of the heliosphere $M_{\infty}\approx 2$ it seems to be a strong argument for the comet-like shape.

Nevertheless, the conclusion cannot be considered final. The next (third) step is to study the bifurcation in the model which includes the interstellar magnetic field. From the first view it seems unrealistic that the interstellar magnetic field may change the critical Mach number from 0.3 to $\sim2$. Nevertheless, the single calculations of the interstellar magnetic field performed by Izmodenov (2018) demonstrate that the heliopause has a tube-like shape for the parameters close to heliospheric (see, e.g. Fig. 3 in Izmodenov 2018). In order to understand how the shape of the heliopause bifurcates depending on the magnitude and direction of the IMF, a detailed parametric exploration is needed. This has not be done so far.

The fourth step consists in exploring how interstellar neutrals influence the critical parameters of the bifurcation. Currently, such exploration has not been done. Single calculations with the realistic heliospheric parameters performed allows us to conclude that the heliosphere has a comet-like shape. However, it is currently unknown how close the actual heliospheric parameters are to the critical parameters of bifurcation.

Finally, we suggest that other groups perform the parametric study in the frame of simplified model as it has been done by Korolkov and Izmodenov (2021). Since numerical reconnection does not appear in this simplified approach, we may expect that all models will give close values of critical Mach numbers at which bifurcation appears. Potentially this allows to identify differences in numerical approaches more easily than by comparing the results of the most complicated multi-component model suites.

## Comparing Heliosphere Models with Measurements: Lessons Learned

From ENA observations at solar wind energy or below obtained with *IBEX* Galli et al. (see 2022) two types of heliospheric ENAs can be distinguished: The globally distributed flux and the so-called Ribbon. The first ENA source shows slow spatial variation, but varies temporally (at least for SW energies or higher) with variations of the SW and ionization rates over the solar cycle, see papers by Galli et al. (2022) and Sokół et al. (2022). The energy spectrum of the globally distributed flux can be described as a sequence of power laws. Below 100 eV the spectrum becomes flatter (e.g. compare Galli et al. 2016 with Zirnstein et al. 2018) and presents two bends around 1 keV and around 5–10 keV. Beyond $\sim5~\text{keV}$ it transitions into energies covered by *Cassini*/INCA ($\sim5.2\text{--}55~\text{keV}$), where the ENA spectra become significantly softer (see Fig. 18; a recent composite spectrum of ENAs and ions within the energy range of $\sim10~\text{eV}$ to 344 MeV in the *V2* direction is shown in Dialynas et al. 2020). The ENA Ribbon, on the other hand, is a $10^{\circ}\text{--}30^{\circ}$ wide feature of enhanced ENA fluxes superimposed on the globally distributed ENA flux. It is most prominent around 1 keV (e.g., at SW energies) and its shape and intensity (not its general location, though) follow the evolution of SW variation over one full solar cycle. As discussed in detail by Galli et al. (2022), the currently favored explanation for the Ribbon are secondary solar wind ENAs (McComas et al. 2020): solar wind protons are neutralized inside the HP, then cross the HP as neutrals, are ionized again, start gyrating around the draped IMF outside the HP and then are neutralized again, thus creating a Ribbon of ENAs just beyond the HP. The 5.2–55 keV ENA images from the Ion and Neutral Camera (INCA) instrument on *Cassini* (Krimigis et al. 2009) placed the local *Voyager* measurements in a global context (Dialynas et al. 2017b), showing the existence of the “Belt,” a high-intensity ENA region of varying emissions and two “Basins” where the ENA minima occur (e.g. Galli et al. 2022; Dialynas et al. 2022).

These ENA observations, together with the in situ ion and magnetic field measurements from the *Voyager* missions, provide invaluable constraints to the various heliospheric models. Most ENA model predictions consistently underestimate the observed ENA intensities over a wide energy range covered with *IBEX* and *Cassini*/INCA from 50 eV to 20 keV (see Galli et al. 2022; Dialynas et al. 2022; Giacalone et al. 2022), as well as Gkioulidou et al. (2022, in press). A part of this underestimation (roughly a factor of two) between observed and predicted ENA intensities could be explained by the hydrogen density at the TS being about 40% higher than assumed in previous models (Dialynas et al. 2019; Swaczyna et al. 2020).

The following paragraphs provide a very brief overview of the lessons we have learned by comparing the recent sophisticated models for the heliosphere, with the various measurements from *IBEX*, *Cassini*, and the *Voyager* missions. Some discussion related to this can also be found in the preceding section.

### Acceleration of ACRs

After reaching the TS, measurements from the *Voyager* missions showed that, contrary to expectations (e.g. Pesses et al. 1981), the high-energy ACRs did not peak at the TS, but continued to increase over a spatial scale of several au deep inside the HS (e.g. Decker et al. 2010; Cummings et al. 2008). These measurements provided no evidence for a possible acceleration of ACRs at the TS. Both *V1* and *V2* observed the so-called “common spectrum” (a power law with a spectral index of −1.5) for particles accelerated to about a few MeV/nuc (Decker et al. 2006; Gloeckler et al. 2008), which was measured downstream of the TS and had the same absolute intensity in both *Voyagers*, despite the fact that the two spacecraft were more than 100 au apart (above and below the ecliptic equator), that remained unchanged for several years after the TS crossings. Furthermore, observations from *V1* (Stone et al. 2013; Krimigis et al. 2013) showed that the ACRs attain their higher energies at distances of greater than $\sim113\text{--}114~\text{au}$ where the spectrum (of, e.g. ACR O, H) is well represented by a power law with a spectral index of −1.5 and an exponential cutoff at $\sim100~\text{MeV}$. Notably, only 20% of the upstream solar wind energy density at the TS went into heating the downstream thermal plasma, while most went into heating the PUI and $>15\%$ went into $>28~\text{keV}$ protons (Richardson et al. 2008; Decker et al. 2008).

These unexpected observations triggered intense discussions among the community to present models that would potentially explain these measurements, with the HS, rather than the TS, being the most likely acceleration region. Some of the proposed mechanisms include the acceleration of ACRs in the presence of a turbulent TS deep in HS (e.g. Kóta 2010; Guo et al. 2010; Giacalone and Decker 2010), the acceleration via magnetic reconnection near the HP (e.g. Drake et al. 2010; Opher et al. 2011), by a turbulent process in the HS (e.g. Fisk and Gloeckler 2009), or acceleration immediately downstream of the TS due to turbulence generated by the coupling of PUIs and multi-ion magnetosonic waves (Zieger et al. 2015). However, Chalov (2005), McComas and Schwadron (2006) posed the hypothesis that ACRs are still accelerated at the TS (in the flanks and tail), as was expected prior to the *Voyager* TS crossings via a “blunt TS geometry,” in which flattening of the nose of the TS leads to a time-dependent acceleration process.

### The Heliosheath Thickness in the Upwind Hemisphere

The width of the HS, i.e. the distance between the TS and HP, was measured to be $\sim28~\text{au}$ toward the *V1* direction and $\sim35~\text{au}$ toward the *V2* direction, i.e. unexpectedly thin. The combined use of *Cassini*/INCA ENAs and *V1/V2* Low Energy Charged Particle (LECP) measurements (Dialynas et al. 2022) gave the opportunity to accurately estimate the HS thickness in both directions (Krimigis et al. 2011; Dialynas et al. 2019), whereas Schwadron et al. (2011, 2014) also approximated the thickness of the HS using *IBEX* ENA measurements. Contrary to observations, most of the current models of the global heliosphere yield thicknesses of the HS that are substantially larger than measured by the *Voyagers*. For example, steady-state kinetic-MHD simulations produce widths for the upwind hemisphere of $>55~\text{au}$ (e.g. Izmodenov and Alexashov 2015). In their work, the authors explain that it is not possible to obtain the TS at a distance of $\sim94~\text{au}$ and the HP at $\sim122~\text{au}$ in *V1* simultaneously in the frame of the global model with the same set of the data-driven boundary conditions. The time-dependent version of the model (Izmodenov and Alexashov 2020) gives the thickness of the heliosheath slightly smaller but still larger than observed.

Adding electron thermal conductivity to their model and under the assumption that the plasma flow in the global heliosphere is nearly isothermal, Izmodenov et al. (2014) were able to obtain excellent agreement between the observed distances to the TS and HP as thermal pressure is decreased in the HS. Pressure can also be removed from the HS via charge exchange, and Heerikhuisen et al. (2008) used a $\kappa $-distribution with $\kappa =1.63$ in all SW plasma (consistent with Decker et al. 2005) to obtain a HS width of $\sim 50~\text{au}$ in the *V1* direction. However, as recently shown by the combination of *Cassini*/INCA remote ENA measurements and in situ ions from LECP, the use of single $\kappa $-distribution underestimates the partial pressure that lies in the 5.2 to 24 keV measurements (Dialynas et al. 2019).

A recent multi-ion MHD model (Opher et al. 2020) predicts a significantly reduced HS width (of the order of $\sim 50~\text{au}$) as compared to the single-ion case ($\sim80\text{--}100~\text{au}$), as a result of charge-exchange losses of the PUIs in the HS, but still the predicted HS thickness in the model is larger than inferred from the observations. Guo et al. (2018) suggested that the thinning of the HS can occur by the loss of a significant fraction of ACRs, whose energy comes from the SW. Borovikov and Pogorelov (2014) suggest that time-dependent Rayleigh-Taylor instabilities at the HP caused by charge exchange could reduce the local HS width down to the observed value. Recently, the HelMod model (Boschini et al. 2019, 2020) toward explaining the evolution of GCRs in the inner and outer heliosphere was able to adequately fit the *Voyager* measurements and obtain their crossings from the TS and HP, including the putative near-TS crossing (Krimigis et al. 2003). Thus, they obtained the correct HS thickness, using a dimensionless stagnation pressure that is not inconsistent with a roughly symmetric diamagnetic heliosphere (Dialynas et al. 2017b).

The discussion of the heliosheath width is complicated by the absence of in situ measurements for the TS and HP distances from the Sun at the same moment of time. The time interval between the TS and HP crossings is about 7.5 and 11 years for *V1* and *V2*, respectively. Pogorelov et al. (2013b), using the boundary conditions for the SW derived from the *Ulysses* data reproduced rather well both the heliocentric distances and times at which *V1* and *V2* crossed. On the other hand, Pogorelov et al. (2015) predicted that *V2* should cross the HP at a distance approximately equal to that of *V1*, which turned out to be rather accurate. A separate treatment of PUIs indeed decreases the width of the heliosheath (Pogorelov et al. 2016). However, data-driven numerical simulations of Kim et al. (2017) and Pogorelov et al. (2021), which involve no special treatment of PUIs, show that this width has been smaller than 40 au since about 2014, reached 30 au in 2017, and now remains almost constant (about 35 au) in the *V1* direction. It was consistently smaller in the *V2* direction, but they became almost identical starting 2017.

### ENA Flux in Global Heliosphere Models

Since 2009, ENA measurements over an extended energy range of $\sim50~\text{eV}$ to $\sim55~\text{keV}$ (McComas et al. 2009; Krimigis et al. 2009) (see also Galli et al. 2022, submitted to ApJS); McComas et al. 2020; Dialynas et al. 2017b, 2020) have provided substantial aid toward constructing global models for the heliosphere and understanding the behavior of PUIs inside the HS, the response of the global heliosphere to the outward propagating SW changes over the solar cycle (SC), and ultimately probing the interaction of the global heliosphere with the VLISM. Recent, sophisticated simulations of $\sim0.71~\text{keV}$ to 4.29 keV ENAs from *IBEX*-Hi (Zirnstein et al. 2017) show a persistent discrepancy between the model and the measurements of a factor of $\sim2\text{--}3$, while in the modeling performed by Baliukin et al. (2022b), where the kinetic treatment of PUIs is utilized, this factor is $\sim1.5$. Although the reasons for this discrepancy are currently not known, Zirnstein et al. (2017) point out that refining the estimations of models for the HS thickness, addressing the velocity diffusion of ions inside the HS (e.g. Fahr et al. 2016), and taking a more precise ion distribution function downstream of the TS (e.g. Dialynas et al. 2020), could perhaps offer an explanation (although other factors could also play a role; see discussion in Zirnstein et al. 2017).

The situation becomes even more puzzling when considering global ENA simulations (Czechowski et al. 2020) at higher energies, where the measured ENA distributions at $> 5.2~\text{keV}$ from INCA (Dialynas et al. 2017a) are higher than the simulations throughout the heliosphere and for both solar minimum and maximum conditions by at least a factor of four, and the measured ENA spectra are much softer than what the simulations predict. Recent analyses employing measurements from various instruments on *Voyager*, *Cassini*, and *IBEX* (Dialynas et al. 2020) over an extended energy range of $\sim10~\text{eV}$ to 344 MeV showed that PUIs and suprathermal particles provide a substantial amount of pressure inside the HS. Under the assumption that all ENAs from both *IBEX* and *Cassini*/INCA originate in the HS, the authors calculated that the overall (isotropic) pressure in the HS in the direction of *V2* is $\sim0.251~\text{pPa}$ (and a plasma $\beta $ of $\sim49$), which is consistent with calculations (Rankin et al. 2019) using data-driven models and observations from *IBEX* (a total effective pressure of $\sim0.267\pm55~\text{pPa}$). Underestimating the flux of ENAs leads to underestimating the partial particle pressure inside the HS (e.g. Dialynas et al. 2019), which is critically important for the determination of the force balance that forms our solar bubble, especially due to the pressure changes inside the HS over the solar cycle. Recently, Krimigis et al. (2019) provided a direct observational verification on the Izmodenov et al. (2008), Izmodenov and Alexashov (2015) time-depended models for the heliosphere, showing that the SW pressure has a large effect on the position of the TS, by as much as 10 au, but minimal effect on the position of the HP, which displays an offset of $\sim3\text{--}4~\text{au}$, despite the substantial changes in the SW pressure at 1 au over the solar cycle.

### Magnetic Field Past the HP

Contrary to theoretical predictions, the crossings of *V1* and *V2* of the HP were associated with no change in the direction of the magnetic field vector (Burlaga et al. 2013, 2019), which remained solar-like regardless of the substantial increases observed in the field magnitude. Notably, the magnetic field intensity and direction past the HP remained nearly constant for several au (e.g Burlaga and Ness 2016) after the crossings, whereas the observed 3–346 MeV H measurements remained consistent with no local interstellar gradient (Cummings et al. 2016).

**Fig. 13:**
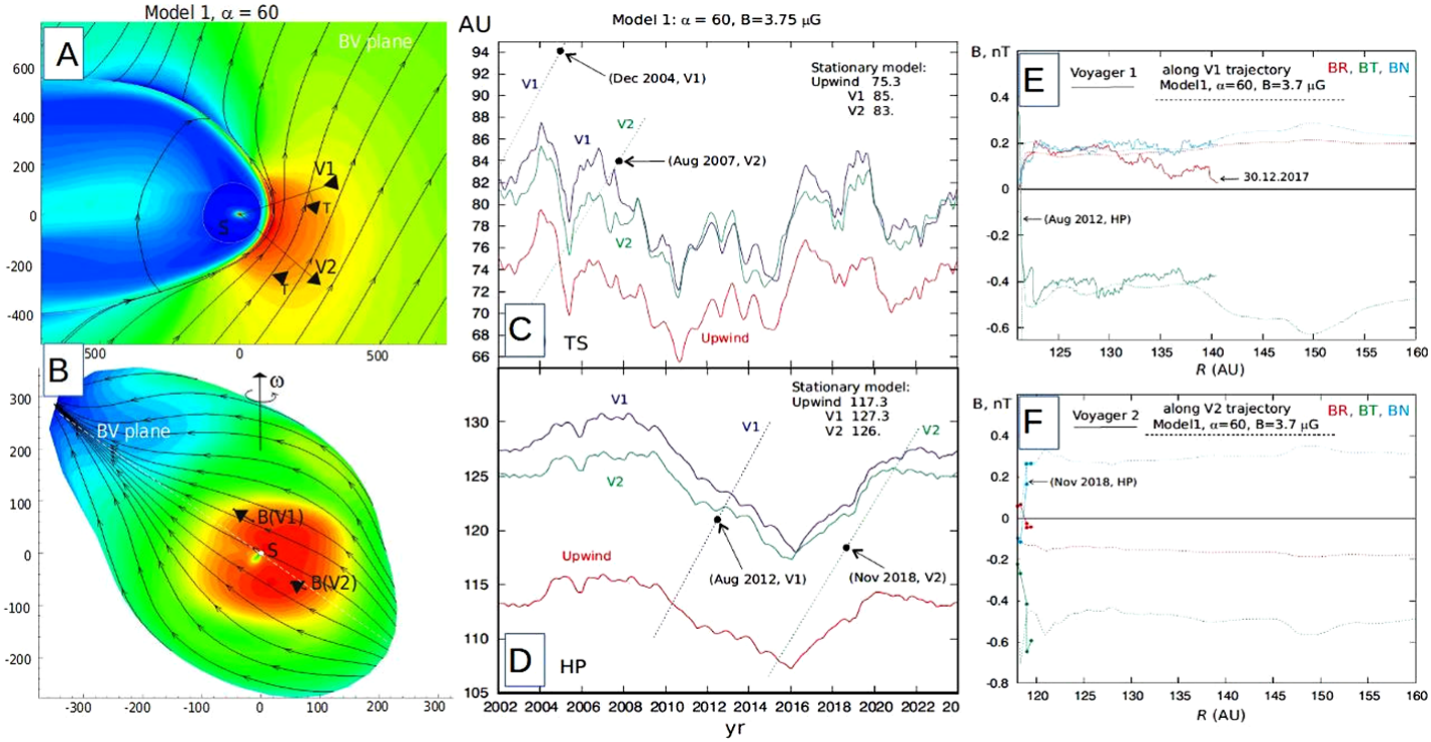
Panel **A**: Magnetic field lines and magnitudes of the magnetic field in the plane determined by $\mathbf{V}_{\mathrm{LISM}}$ and $\mathbf{B}_{\mathrm{LISM}}$ vectors. Panel **B**: Projections of magnetic field lines on the heliopause. Panels **C** and **D**: Heliocentric distances to the TS (panel **C**) and HP (panel **D**) are shown for the directions of *V1* (blue curves), *V2* (green curves) and in the upwind direction (red curves). Panels **E** and **F**: Components of the magnetic field in the RTN coordinate system along the *V1* (panel **E**) and *V2* (panel **F**) trajectories. The model results are shown as dashed curves. Solid curves represent *V1*/MAG data in panel **E** and *V2*/MAG data in panel **F**. The model curves are shifted by 3 au in panel **E**, and by 5 au in panel **F**. After Izmodenov and Alexashov (2020)

Pogorelov et al. (2021) specifically addressed the issues related to the magnetic field behavior observed by *V1* and *V2* when they crossed the HP. In particular, it has been shown that the assumption of unipolar HMF results in solutions that contradict observational data: the magnetic pressure is continuous across the HP and reaches its maximum at the HP itself, while the magnetic field direction is different on its SW and LISM sides. However, the asymptotic magnitude and direction behind the HP were still adequately reproduced under this assumption in Pogorelov et al. (2017b, 2021) and Opher et al. (2020), where the magnetic field vector in the unperturbed LISM is directed approximately (Zirnstein et al. 2016) towards the center of the *IBEX* ribbon, which is known to form from secondary charge-exchange interactions beyond the HP. On the other hand, the data-driven simulations presented by Pogorelov et al. (2021) exhibit jumps in the magnetic pressure and very small magnetic field rotation. It is also acknowledged in that paper that this outcome cannot be universal, since the HMF changes its polarity each solar cycle, while the ISMF direction does not. Data-driven simulations presented in Kim et al. (2017) and Pogorelov et al. (2021) show the development of shocks that propagate through the VLISM and their interaction consistently with the *Voyager* data. Izmodenov and Alexashov (2020) show that just beyond the HP, all three components of the IMF obtained in their data-driven model match those measured by both Voyagers (see Fig. 13).

**Fig. 14:**
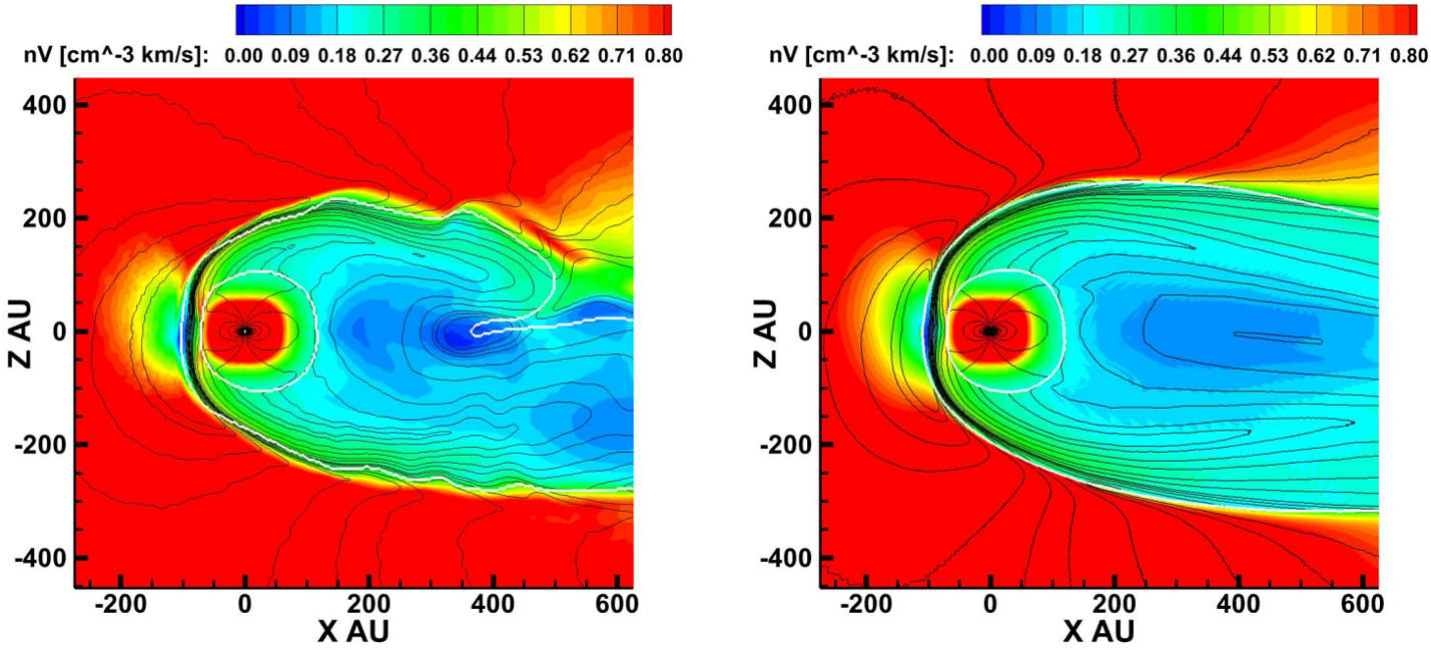
Meridional slices of the BU (left) and Moscow (right) models of the heliosphere showing the mass flux ($n_{\mathrm{p}}V_{\mathrm{p}}$) through the HS. Black lines represent the magnetic field intensity overlaid on the mass flux and white lines represent the HP. The HP is approximated after the solution is obtained by an isosurface of solar wind density at $0.005~\text{cm}^{-3}$. This isosurface is chosen such that only purely solar magnetic field lines are within and reconnected field lines draping around the HP. The mass flux in the HS for both models is organized by the solar magnetic field. The coordinate system is such that the $z$-axis is parallel to the solar rotation axis and the $x$-axis is $5^{\circ}$ above the direction of interstellar flow, with $y$ completing the right-handed coordinate system. Adapted from Kornbleuth et al. (2021b)

**Fig. 15:**
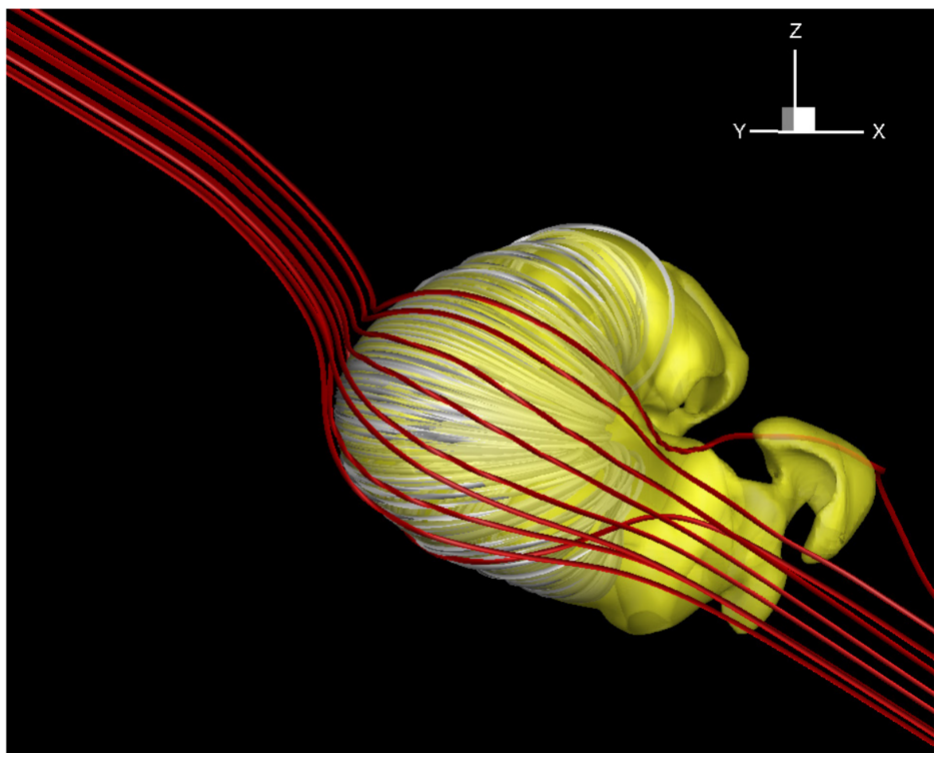
A deflated heliosphere when the PUIs are treated as a separate component than the ionized component (Opher et al. 2020). 3D view of the heliosphere. The yellow isosurface reflects the heliopause (which is found as stated in the caption of Fig. 14), the white lines reflect the solar magnetic field, and the red lines reflect the draped interstellar magnetic field

**Fig. 16:**
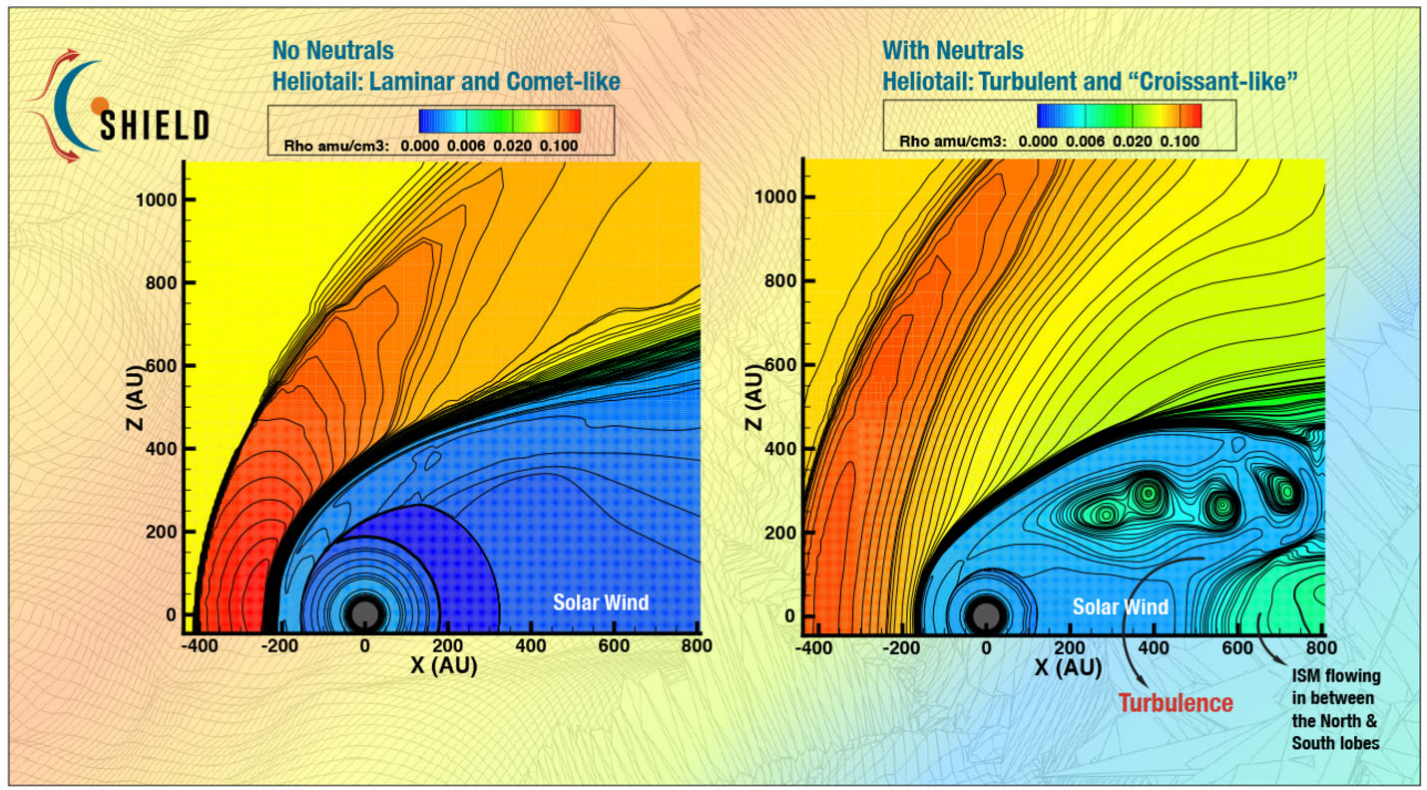
Plots adapted from Opher et al. (2021) showing the effect of charge exchange between the SW plasma and neutral H. Charge exchange results in a Rayleigh-Taylor-like instability, which opens the heliotail. Left: meridional slice of the BU model without charge exchange between the solar wind plasma and interstellar neutrals. Right: meridional slice of the BU model with charge exchange included between the solar wind plasma and interstellar neutrals. Coordinate axes are oriented as in Fig. 14

**Fig. 17:**
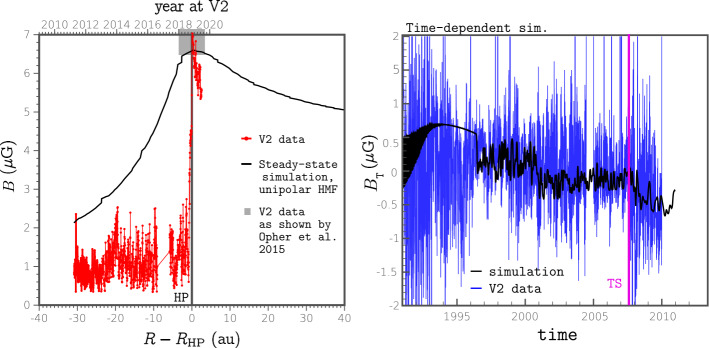
Comparison of *V2* data and simulation results. Left panel: Distributions of magnetic field strength along the *V2* trajectory in the SW–LISM interaction assuming unipolar heliospheric magnetic field. The simulation grid size is $<1~\text{au}$ in all directions. Adapted from Pogorelov et al. (2021). The gray rectangle is copied from Opher et al. (2020), where it is claimed that *V2* data near the HP remain within this rectangle. Right panel: Distributions of the T-component of the HMF vector from the time-dependent calculation of Pogorelov et al. (2013a) based on *Ulysses* data

**Fig. 18:**
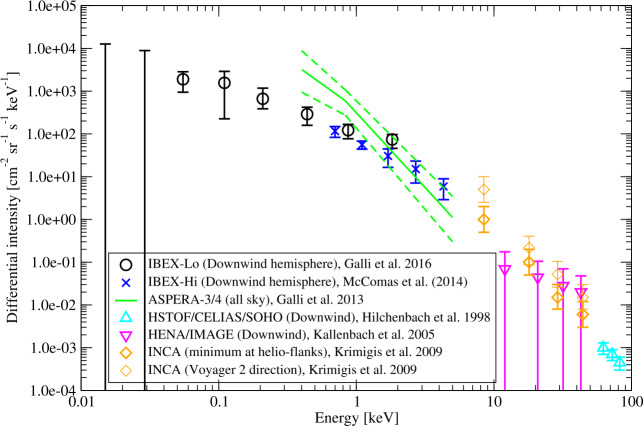
Compilation of heliospheric ENA spectra for some sky regions from all ENA observation data sets so far available over the complete observed energy range

The possibility that the observed magnetic field direction is due to the location and geometry of the *Voyager* trajectory (e.g. Grygorczuk et al. 2014) may not provide an adequate explanation for this enigmatic feature.

### Low-Energy and GCR Measurements Past the Heliopause

The crossings of *V1* and *V2* of the HP were associated with a gradual decrease of the number of energetic ions of solar origin within a few au, and an overall increase of ions of Galactic origin (GCR) by $\sim30\%$ in *V1* and $\sim20\%$ in *V2*, over a period of a few weeks before an abrupt change that occurred in the HP region (Krimigis et al. 2013, 2019; Stone et al. 2013, 2019). Contrary to theoretical expectations, where the GCRs in the upstream VLISM would be isotropic, $>211~\text{MeV}$ measurements from LECP showed several episodes of reduced proton intensity and time-varying depletion of particles with pitch angle close to $90^{\circ}$ (Krimigis et al. 2013; Hill et al. 2020). Toward explaining these episodic (anisotropic) reductions of GCRs upstream into the VLISM, Roelof et al. (2013) suggested that *V1* surveyed a region of weaker magnetic field, where adiabatic focusing produced a narrow gap in the distribution of GCRs near $90^{\circ}$ pitch angle. In a similar interpretation that focused on the time evolution of the events, rather than solely the spatial variations (as in Roelof et al. 2013), the numerical simulations from Jokipii and Kóta (2014) and Kóta and Jokipii (2017) suggested that these episodes are a result of adiabatic cooling behind a shock (that reached the magnetic field line passing through *V1*) due to the slow weakening of the magnetic field.

Of particular interest is that the LECP observations on *V1* showed that the HP may be a region that involves an interchange instability (Krimigis et al. 2013) at the boundary. Simulations by Florinski (2015) and Florinski et al. (2015) verified that during the interchange instability the flux tubes are connected to the VLISM at both ends and energetic particles can escape in both directions with equal probability, whereas the remaining gyrating particles can develop a second-order anisotropy. This model can also provide an adequate explanation for the recent LECP observation of low-energy ions ($\sim40\text{--}139~\text{keV}$) from the HS streaming out to the VLISM for $\sim28~\text{au}$ past the HP (Dialynas et al. 2021). However, a different interpretation for these measurements may indicate that *V1* is still surveying the HS, which contradicts previous interpretations of the *V1* measurements (e.g. Burlaga et al. 2013; Krimigis et al. 2013; Stone et al. 2013), as predicted by Fisk and Gloeckler (2014), showing an essentially constant outflow of low-energy energetic particles in the radial direction, up to $\sim150~\text{au}$. Unlike *V1*, which found two interstellar flux tubes that had invaded the HS with strong anticorrelations in GCRs, *V2* found no similar precursors to the HP (Stone et al. 2019).

## Summary and Conclusions

In this paper, we have presented a review of a (hopefully representative) subset of the methods employed to model the properties of the global heliosphere and its interaction with the interstellar matter through which it moves, and of the results which these works have produced so far. The first decades of heliospheric research were, quite naturally, dominated by theoretical considerations which relied on analytical methods, and many of the concepts and findings from this stage continue to be relevant today. Our knowledge of the heliosphere’s physical properties received a major boost when in situ observations from the *Voyager* spacecraft became available, and another one through the more recent exponential rise in computing power and storage capacity that made large grid-based simulations possible. As a result, the field of heliophysics is now very much dominated by large, high-performance, numerical modeling. This methodological shift is, to some extent, also reflected in the layout of this paper, with its single section on past and current analytical models and a total of five sections exclusively dealing with numerical works.

Although the complexity required to explain the intricate details of the contemporary wealth of heliospheric observations can arguably be appropriately addressed only with equally complex simulations, analytical solutions are not just intellectually rewarding but also useful for simpler or more general considerations, where the full computational machinery is either not available or not deemed necessary. This may lend support to the notion that the interplay between and the complementary nature of analytical and numerical modeling is well-suited to advance our understanding of the heliosphere in which we live.

## Footnotes

1 The original flow field derived by Macquorn Rankine, which he developed to model the flow of water around sea-going vessels, uses an additional sink of strength $-4\pi u_{0} q$ trailing at a finite distance behind the source.

2 Hierarchical Data Format, see http://www.hdfgroup.org/solutions/hdf5.

3 https://sites.bu.edu/shield-drive/.
